# *HYR1*-Mediated Detoxification of Reactive Oxygen Species Is Required for Full Virulence in the Rice Blast Fungus

**DOI:** 10.1371/journal.ppat.1001335

**Published:** 2011-04-14

**Authors:** Kun Huang, Kirk J. Czymmek, Jeffrey L. Caplan, James A. Sweigard, Nicole M. Donofrio

**Affiliations:** 1 Department of Plant and Soil Sciences, University of Delaware, Newark, Delaware, United States of America; 2 Department of Biological Sciences, University of Delaware, Newark, Delaware, United States of America; 3 Delaware Biotechnology Institute, Newark, Delaware, United States of America; 4 Stine-Haskell Lab, DuPont, Newark, Delaware, United States of America; University of Melbourne, Australia

## Abstract

During plant-pathogen interactions, the plant may mount several types of defense responses to either block the pathogen completely or ameliorate the amount of disease. Such responses include release of reactive oxygen species (ROS) to attack the pathogen, as well as formation of cell wall appositions (CWAs) to physically block pathogen penetration. A successful pathogen will likely have its own ROS detoxification mechanisms to cope with this inhospitable environment. Here, we report one such candidate mechanism in the rice blast fungus, *Magnaporthe oryzae,* governed by a gene we refer to as *MoHYR1*. This gene (MGG_07460) encodes a glutathione peroxidase (GSHPx) domain, and its homologue in yeast was reported to specifically detoxify phospholipid peroxides. To characterize this gene in *M. oryzae*, we generated a deletion mutant*Δhyr1* which showed growth inhibition with increased amounts of hydrogen peroxide (H_2_O_2_). Moreover, we observed that the fungal mutants had a decreased ability to tolerate ROS generated by a susceptible plant, including ROS found associated with CWAs. Ultimately, this resulted in significantly smaller lesion sizes on both barley and rice. In order to determine how this gene interacts with other (ROS) scavenging-related genes in *M. oryzae*, we compared expression levels of ten genes in mutant versus wild type with and without H_2_O_2_. Our results indicated that the *HYR1* gene was important for allowing the fungus to tolerate H_2_O_2_ *in vitro* and *in planta* and that this ability was directly related to fungal virulence.

## Author Summary

Reactive oxygen species (ROS) are antimicrobial compounds and also serve as stimulators and products of plant defense reactions. ROS appear to be active in the critical zone where pathogens and plants come in contact. Therefore, understanding the source, the role, and the destination of ROS in each interacting partner will be crucial for understanding the pathogen-host molecular battle. In this study, we focused on one potential fungal mechanism for ameliorating effects of plant-produced ROS during the early stages of infection. Characterizing the *MoHYR1* gene from the rice blast fungus *Magnaporthe oryzae*, suggested that *MoHYR1* was involved in overcoming plant defense-generated ROS. The deletion of this gene caused a virulence defect in *M. oryzae*, which we believe was associated with the mutant's inability to detoxify plant-generated ROS. Together, our data suggested that HYR1 is a virulence factor in the rice blast pathogen, and its role in virulence was directly related to sensing and managing plant-generated ROS during early infection events. HYR1 is part of a ROS scavenging and sensing pathway that is well characterized in yeast, and our study is the first to examine this important gene in filamentous fungi.

## Introduction

Molecular oxygen, itself relatively nontoxic, is important to most living organisms on this planet. However, its derivatives, reactive oxygen species (ROS), can lead to oxidative destruction of cells [1]. For example, in mammals, ROS can accelerate aging by making holes in membranes, or by stealing electrons from DNA, which may result in cancer and other severe diseases [2]. However, animals, plants and fungi have all adapted to use ROS as key signaling molecules [3]. In plants, ROS play a more positive role as a defense mechanism against attacking pathogens, and are often produced as a first line of defense [4]. In the plant-pathogenic fungus, *Magnaporthe oryzae*, ROS regulation plays important roles in both development and virulence. ROS itself has been shown to accumulate in the developing and mature appressorium, or fungal penetration structure, while the two NADPH oxidases in *M. oryzae*, *NOX1* and *NOX2* are required for proper development of appressoria, as well as full virulence [5]. The catalase gene family member, encoded by *CATB*, was shown to also be involved in cell wall integrity as well as virulence, as deletion mutants were altered in hyphal, spore and appressorial morphology [6]. Organisms, therefore, must carefully balance the toxic effects of ROS and the need for ROS in cellular signaling.

There are five major types of ROS in plants: superoxide (O_2_^−^), hydrogen peroxide (H_2_O_2_), hydroxyl radical (OH), nitric oxide (NO), and singlet oxygen (^1^O_2_). In plant cells, organelles with an intense rate of electron flow or high oxidizing metabolic activity are major sources of ROS generation [7]. These organelles include mitochondria, chloroplasts and peroxisomes. ROS are also generated via enzymatic sources, such as membrane-associated NADPH oxidases, cell wall peroxidases and oxalate oxidases [8].

ROS play a crucial role during plant defense responses. Oxidative bursts have been detected when plant cells are inoculated with biotrophic pathogens [9], hemi-biotrophic pathogens [10], necrotrophic pathogens [11], and pathogen elicitors [12]. More recent studies with *M. oryzae* that causes rice blast disease, demonstrated that rice produces H_2_O_2_ shortly after inoculation with a virulent strain of the fungus [13], [14]. The toxic effects of ROS can directly kill pathogens, and as a result, pathogens have developed counter measures [5]. The coexistence of hosts and pathogens side-by-side determines that the increase of resistance in a host will be balanced by the change of virulence in a pathogen, and vice versa. A metabolite fingerprint study of three rice cultivars infected by *M. oryzae* provided evidence for suppression of plant-associated ROS generation during compatible interactions [9]. Fungal-produced catalase was secreted during infection, and appeared to play a role in breaking down the plant-produced H_2_O_2_, allowing the disease cycle to occur; in the absence of catalase, infection was largely blocked by the plant's ROS [15].

ROS production and mitigation is a multifaceted process, incorporating many genes and pathways [1]. One mechanism of sensing and ultimate detoxification of ROS in yeast is via the *Hyr1* gene, formerly termed *Gpx3*/*Orp1*; this gene, upon ROS induction, activates its partner protein yAP1, which is a bZip transcription factor involved in activating cellular thiol-redox pathways, and arguably one of the most studied ROS-sensing proteins in yeast [16]. This AP1-like (activator protein) transcription factor regulates H_2_O_2_ homeostasis in *Saccharomyces cerevisiae* (*S. cerevisiae*), which in turn governs the synthesis of glutathione [17]. Hyr1p plays a key role during the oxidative response in *S. cerevisiae* [18]; after being directly oxidized by H_2_O_2_, it forms an intermolecular disulfide bond with yAP1 [19]. A conserved cysteine residue at position 598 in Yap1p becomes active by forming an inter-molecular disulfide bond with the Cys36 of Hyr1p. This transient inter-molecular linkage is then resolved to a Yap1p intra-molecular disulfide bond between the cysteines at positions C303-S-S-C598. During this process, the Yap1 protein is released by Hyr1p in its active form, which is then transported to the nucleus [20]. This conformational change shields its nuclear export signal from the interacting protein Crm1p, allowing it to remain in the nucleus and control a suite of antioxidant genes [21], [22]. Although *YAP1* gene homologs have been analyzed in several plant pathogenic fungi such as *Aspergillus fumigatus, Alternaria alternata, Cocholiobolus heterostrophus, Botrytis cinerea* and *Ustilago maydis* [16], [20], [23], [24], [25], [26], *HYR1* has yet to be studied in filamentous fungi.

In this study, we closely examined the *HYR1* homolog in *M. oryzae* as a candidate mechanism for coping with a ROS-intensive host environment. We demonstrated that *HYR1* was indeed involved in detoxifying or preventing plant basal immune responses including plant-generated ROS and callose deposits during initial stages of infection, which was correlated with its role as a virulence factor.

## Results

### Identification and characterization of a Glutathione peroxidase domain-containing gene in the genome of *M. oryzae*

**Figure 1:**
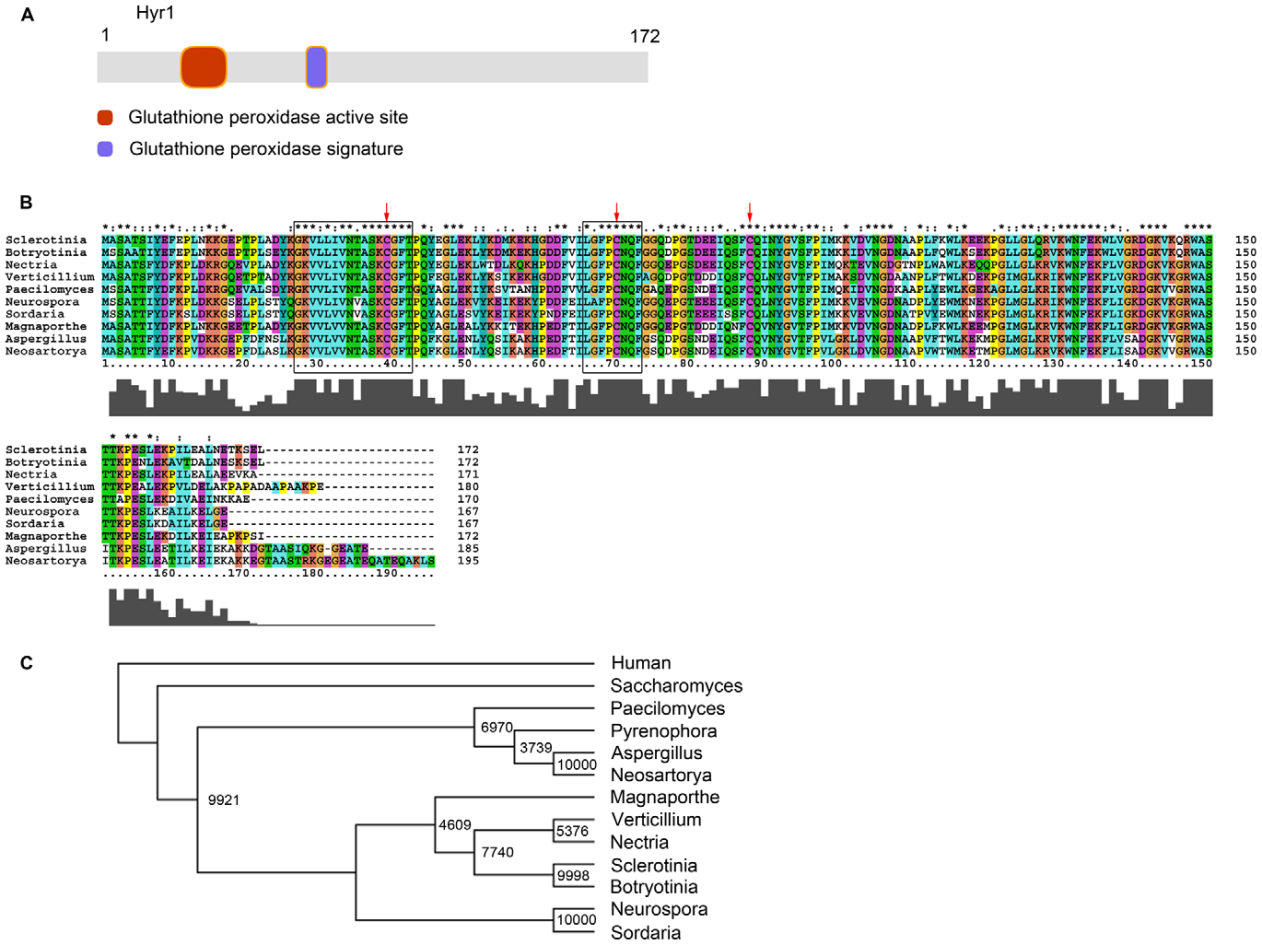
MoHYR1 is a putative thioredoxin peroxidase protein and highly conserved among filamentous fungi. (**A**) A Prosite search of the amino acid sequence revealed two glutathione peroxidase domains, the first of which is an active site, and the second, a signature (image was drawn with DomainDraw, [45]). (**B**) Alignment of the *M. oryzae* HYR1 with nine filamentous fungi. Shaded boxes below the alignment indicate degree of conservation. Open boxes indicate locations of domains in A. Arrows indicate the conserved cysteines. (**C**) Dendrogram of HYR1 from eleven filamentous fungi, one copy from yeast and one from human.

**Figure 2:**
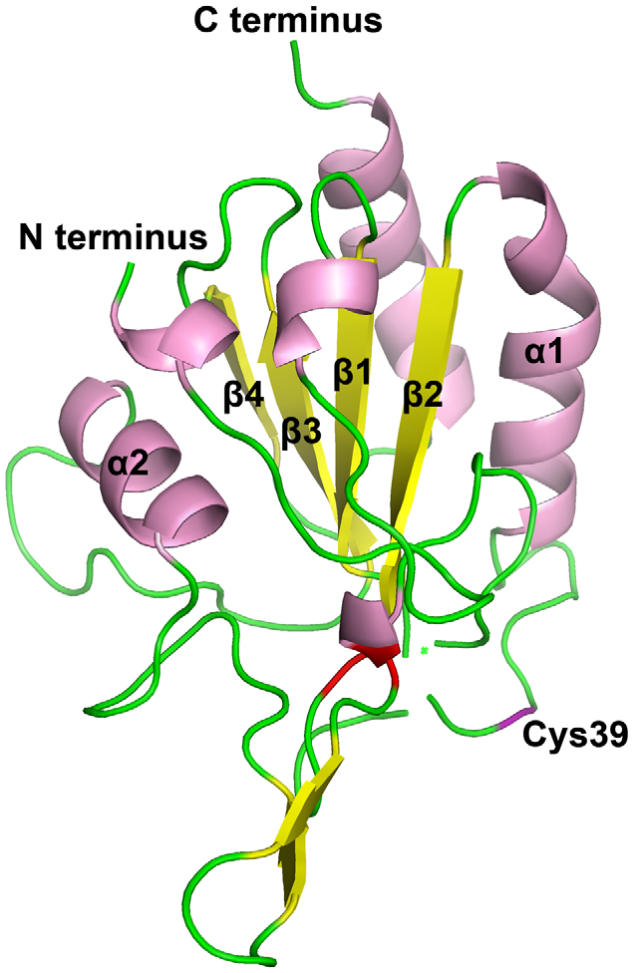
*M. oryzae* HYR1 shares similar tertiary structure with yeast HYR1. The predicted tertiary structure of MoHYR1 from *M. oryzae* was constructed with the PyMOL program. Helices, sheets and termini are tentatively labeled according to the yeast HYR1 structure; the two connecting cysteines are in red, while the cysteine (Cys 39) that would form an intermolecular bond with the HYR1-interaction protein, YAP1, is shown in purple and labeled.

As one of the key members during the oxidative stress response, the yeast *Saccharomyces cerevisiae* Hyr1/YIR037W (formerly termed Gpx3) was reported to be a glutathione-dependent phospholipid peroxidase (PhGpx) that specifically detoxifies phospholipid peroxides [19]. In order to identify the corresponding gene in *M. oryzae*, we performed a BlastP analysis against the fully sequenced genomic database of *M. oryzae* housed at the Broad Institute. Using an E-value of 1e-3 returned a single hit located on Supercontig 20, with an accession number of MGG_07460.6. It is 1315 bp long including two introns, with an open reading frame of 783 bp, which encodes a 172-amino acid protein. A sequence analysis was performed using Prosite on the ExPASy Proteomics Server (http://ca.expasy.org/prosite/). Hits revealed a glutathione peroxidase active site at amino acid positions 27–42, and a glutathione peroxidase signature at amino acid positions 66–73 (Figure 1A). When a BlastP search was performed against GenBank at NCBI, numerous hits were returned with high similarity scores, from many organisms including fungi and bacteria. An alignment indicates that the putative GSHPx domains of Hyr1 are highly conserved across different organisms (Figure 1B). The MoHyr1 protein shares the highest amino acid conservation with the model, non-pathogenic fungus, *Neurospora crassa* (93% similarity and 73% identity), but shares between 81 and 90% similarity with eight other plant pathogenic filamentous fungi examined (Table S1 and Figure 1C). Secondary structure of the HYR1 protein was determined by PSIPRED [27], and consists of eight β-sheets (or strands) and four α-helices (Figure 2). As described in Zhang et al [18], the ScHyr1p showed a typical ‘thioredoxin fold’, also consisting of four β-sheets surrounded by three α-helices [28]. We compared the crystal structure of ScHyr1p with the predicted tertiary structure of MoHyr1 protein, generated with PyMOL (http://www.pymol.org/). The MoHyr1 predicted structure appears similar to a canonical thioredoxin fold, showing four β-sheets, with β1 and β2 running parallel and β3 and β4 running anti-parallel, surrounded by three α-helices (Figure 2). We located three positionally conserved cysteines in our HYR1 protein model compared to yeast, and these are marked in Figures 1B and 2. Two important cysteines, Cys39 and Cys88, likely correspond with two active sites found in the yeast Hyr1p, Cys36 and Cys82. Together, our *in silico* data suggest that we have identified the structural homolog of the ScHyr1 from yeast, and that this gene is highly conserved across filamentous fungi.

**Figure 3:**
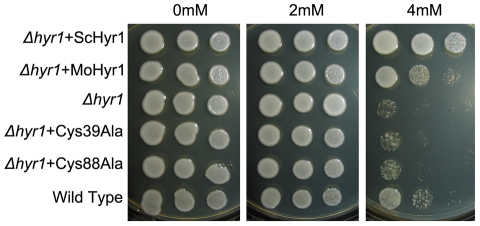
*MoHYR1* complements the *S. cerevisiae Δhyr1* mutant. The yeast strains BY4741 (wild type) and BY4741 YIR037W (*Δhyr1*) were obtained from the ATCC. The mutant was complemented with the wild type copy of itself, the *MoHYR1* gene, and the *MoHYR1* containing mutations at each of the two cysteine residues (cys39Ala and cys88Ala). All strains were spotted onto YPD plates containing 0 mM, 2 mM and 4 mM hydrogen peroxide. Neither the YIR037W strain, nor the two cysteine residue mutants grow at the non-permissive concentration however the yeast mutant is partially rescued by the *MoHYR1* copy. This experiment was repeated ten times with similar results.

In order to functionally characterize the *MoHYR1* gene, we obtained the ATCC *S. cerevisiae Δhyr1* mutant and its wild type parent for complementation tests. Our hypothesis was that based on its sequence and predicted tertiary structure, the *MoHYR1* gene would rescue the yeast mutant when grown on non-permissive concentrations of hydrogen peroxide. As shown in Figure 3, the yeast mutant and the wild type strain both grow well on 0 and 2 mM H_2_O_2_. However, growth of yeast *Δhyr1* was significantly hindered in 4 mM H_2_O_2_. The wild type *MoHYR1* gene was transformed into the yeast mutant, which restored partial growth on this higher concentration. To further support our hypothesis, we constructed mutations in the two conserved cysteine residues at positions 39 and 88. Neither of the mutations rescued the yeast phenotype on hydrogen peroxide (Figure 3).

### Targeted deletion of *MoHYR1*

To explore the biological role of the MoHyr1 protein in the development and pathogenicity processes of *M. oryzae*, the deletion mutant *Δhyr1* was generated through homologous recombination of the *MoHYR1* open reading frame with a gene conferring hygromycin resistance (hygromycin phosphotransferase; HPH) (Figure S1A). A gene deletion fragment was generated by nested PCR amplification of the 5′ flanking region of *MoHYR1*, the *HPH* gene, and 3′ flanking region of *MoHYR1*, using adapters to link the three pieces together. This gene deletion fragment, which contained flanking regions homologous to the *MoHYR1* gene, was introduced into protoplasts of *M. oryzae* via PEG-mediated fungal transformation. After PCR screening of successful knockouts and ectopics using primer pairs outside the flanking regions and inside the HPH gene, two *Δhyr1* knockout mutants (B25, B33) and two ectopic mutants (B40, B60) were identified (Figure S1B) and confirmed with Southerns (Figure S1C). Real-time qRT-PCR was also employed to confirm full deletion of the *MoHYR1* gene and no transcripts were detected. Deletion mutant *Δhyr1* (B33) was complemented with a full-length copy of the *MoHYR1* gene linked to the cerulean fluorescent protein (Figure S1D, see Materials and Methods).

### MoHYR1 is required for vegetative hyphal growth in a ROS-rich environment

HYR1p in yeast was reported to not only be a sensor of ROS, but to have scavenging properties as well [19]. To investigate the role of MoHYR1 in scavenging H_2_O_2_ during vegetative hyphal growth, we inoculated the same amount of initial mycelia into complete media (CM) containing 0, 5 and 10 mM H_2_O_2_. No significant differences were detected among wild type, the *Δhyr1* knockout mutants and the ectopics when growing in 0 mM H_2_O_2_. However, the mycelial growth of the *Δhyr1* knockout mutants was severely and significantly affected at 10 mM H_2_O_2_ (Figure S2A and B). By contrast, the wild type and ectopics did not display much difference in mycelial growth at any concentration. The complemented mutant line grew slightly better than wild type in all concentrations of H_2_O_2_, and upon Southern analysis, we found that four copies had inserted into the genome (Figure S1E). Together, these data indicated that *MoHYR1* was responsible for the H_2_O_2_ growth tolerance phenotype.

### The *MoHYR1* gene contributes to virulence in *M.oryzae*

**Figure 4:**
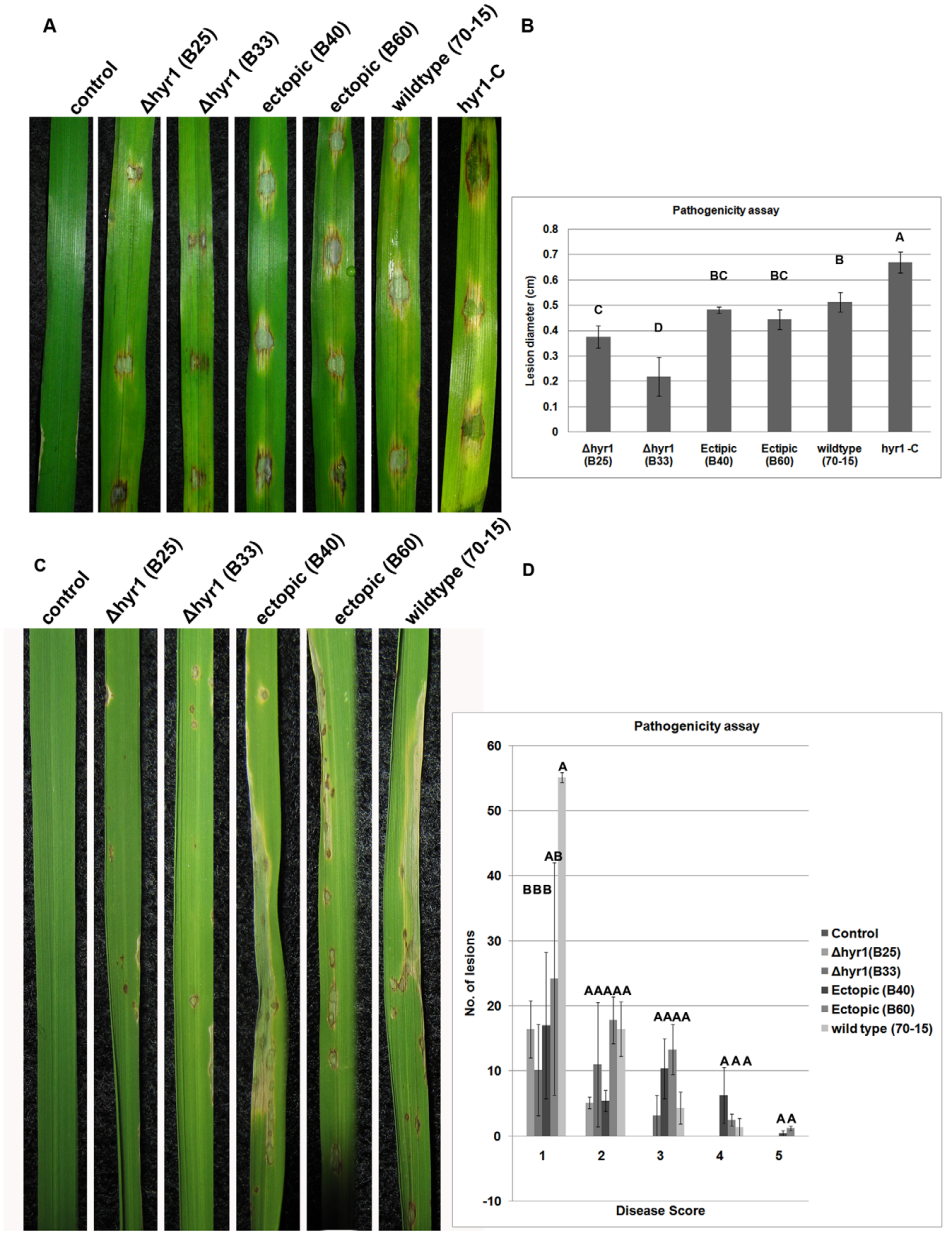
*Δhyr1* exhibits a virulence defect. Δ*hyr1* mutants display a decrease in pathogenicity compared to wild type, on susceptible barley and rice. (**A**) Conidia of two Δ*hyr1* mutants, B25 and B33, were drop-inoculated onto barley cultivar Lacey and show a virulence defect compared to ectopics (B40 and B60), the complemented line (*Δhyr1 - C),* or 70-15 (wild type), as manifested by smaller lesions at 7dpi. (**B**) Quantification of lesion size reveals a significant difference in virulence between wild type and ectopics, and the mutants. Different letters over the bars indicate a significant difference as determined by a student's t-test, and a p-value of ≤0.01. (**C**) Rice plants (cultivar Maratelli) were spray-inoculated with the mutants, ectopics and wild type (as above) and scored for lesion type 7 dpi. (**D**) Quantification of lesion type (0 = no symptom; 1 = pinhead-sized brown specks; 2 = 1.5 mm brown spots; 3 = 2–3 mm gray spots with brown margins; 4 =  many elliptical gray spots longer than 3 mm; 5 = coalesced lesions infecting 50% or more of the leaf area), reveals no difference in lesion types 1-3 however the two mutants do not make any lesion types 4 and 5. Lesions were photographed and measured or scored 7dpi and experiments were repeated twice with similar results. Different letters over the bars indicate a significant difference as determined by a student's t-test and a p-value of <0.05.

To determine the role of *MoHYR1* in virulence, we drop-inoculated detached leaves of three week-old blast-susceptible barley cultivars with conidia from two independently generated *Δhyr1* mutants, B25 and B33 (Figure 4A). The mutants were still able to cause disease lesions, but there was a measurable and significant reduction in lesion size compared to those produced by wild type, ectopics, and the complemented line (Figure 4B). The complemented line, *hyr1*- C, restored full virulence to the *Δhyr1* mutant, B33. All pathogenicity assays were repeated on the susceptible rice cultivar Maratelli, with similar results (Figure 4C) using the spray-inoculation technique. Disease was also quantified on rice using a “lesion type” scoring assay [29] and error bars show that while lesion types 1–3 do not differ between the mutants, ectopics and wild type, lesion types 4 and 5 (severe, coalescing) did not form on mutant-inoculated plants (Figure 4D) Interestingly, no other developmental phenotype examined was compromised in the *Δhyr1* mutant, including growth rate, conidia production and shape, germ tube and appressorial formation (Table 1).

**Table 1:** Development characteristics of the *Δhyr1* mutant are similar to ectopics and wild type.

	Growth rate (cm)	Conidiation^1^	% GT^2^ formation	% AP^3^ formation	Conidia shape
Strain					
70-15 (WT)	5.03+0.32	21.33+11.06	0.91+0.08	0.93+0.06	normal
*Δhyr1* mutant	5.13+0.06	19+1.73	0.95+0.09	0.92+0.02	normal
Ectopic	4.9+0.44	20+0	0.93+0.08	0.97+0.05	normal

1 concentration equals 1×10^5^conidia/ml.

2 GT  =  germination tube.

3 AP  =  appressorium.

### MoHyr1 is required for breaking down ROS *in planta* during infection but not for internal ROS levels

**Figure 5:**
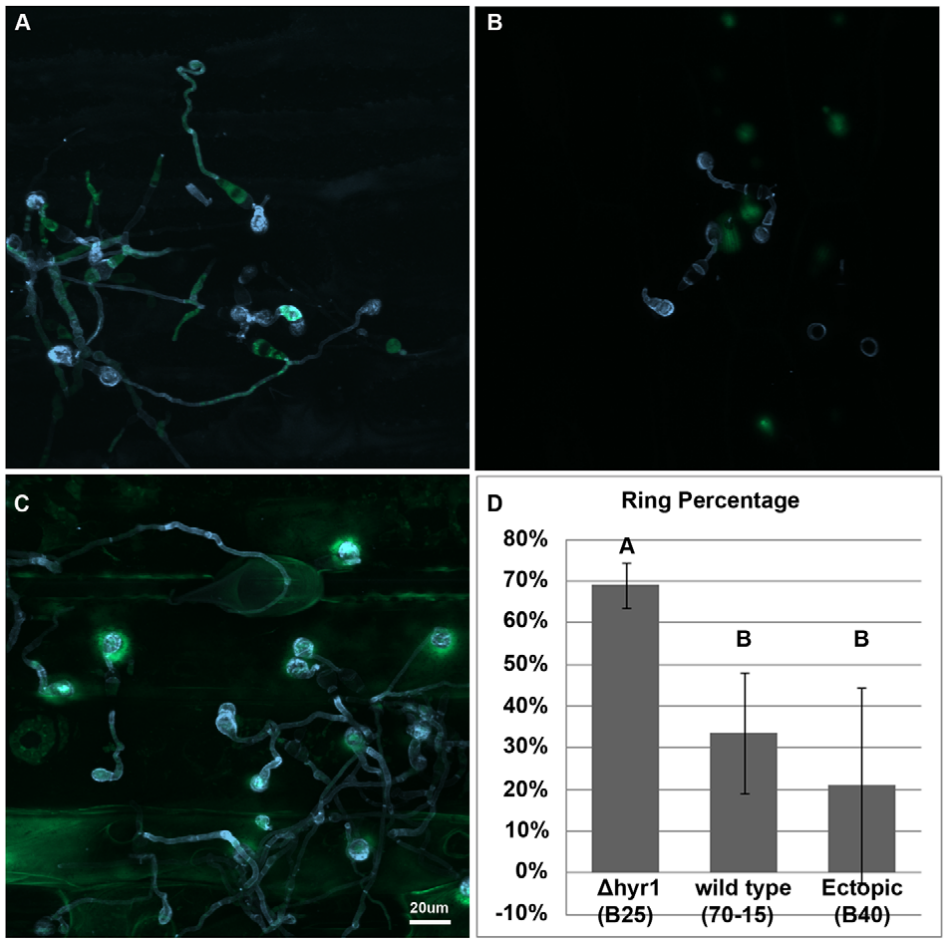
More ROS was produced when leaves were inoculated with *Δhyr1* mutant conidia, versus wild type. (**A**) Conidia of wild type (70-15), (**B**) ectopic (B40) and (**C**) *Δhyr1* (B25) were inoculated onto the surface of a barley leaf and then stained with calcofluor white for fungal cell walls and DCF for the ROS, 24 hpi and imaged by confocal microscopy. (**D**) Around 35 Appressoria were counted for each line, along with the number of appressoria showing ROS haloes, and percentages were generated. This experiment was repeated ten times with similar results. Different letters over the bars indicate a significant difference as determined by a student's t-test, and a p-value of ≤0.05. Scale bar  = 20 µm for all images.

A fundamental question we wanted to assess was whether MoHYR1 was required for infection-related activities *in planta*. The *M. oryzae*'s disease cycle is initiated when the conidium contacts a hydrophobic surface, inducing it to germinate. The germinated conidium forms a germ tube and appressorium that penetrates the plant surface via turgor pressure and forms a thin penetration peg into the first plant cell [30]. Thus, we first examined whether ROS was present during any of these processes, and if so whether MoHYR1 was involved in coping with it. We inoculated susceptible rice and barley cultivars with the *Δhyr1* mutants, ectopics and wild type. ROS was detected using the indicator 2′,7′-dichlorodihydrofluorescein diacetate (H_2_DCFDA) [31]. Conidia of wild type, ectopics and the *Δhyr1* mutant all elicited some degree of ROS when inoculated onto barley leaves (Figure 5A–C), whereas ROS was undetectable under the same imaging conditions when non-inoculated leaves were stained (data not shown). The *Δhyr1* mutants showed the strongest ROS signal 24 hours post inoculation (hpi) compared to the others. The signal continued in this manner through 48 hours (data not shown). These experiments were repeated six times and the results were consistent across the two independent *Δhyr1* mutant lines. ROS signals were quantified via counting the number of ‘ROS haloes’ found around appressoria and expressing this as a percentage of appressoria counted per sample; a significant difference in signals was observed between the mutants, wild type, and ectopics (Figure 5D). These results indicate that in the absence of the *MoHYR1* gene, the fungus can no longer manage the ROS that is generated during initial infection events, or loses the ability to effectively cope with it.

To better understand the reason for reduced virulence in the *Δhyr1* mutant, we wished to determine whether internal fungal levels of ROS were altered in the absence of the gene. The deletion mutant and wild type were grown on complete media and stained with nitroblue tetrazolium (NBT) for production of superoxide anions (Figure S3). Results showed little differences between mutant and wild type when examining the entire colony (Figure S3E and F) or aerial hyphae (Figure S3A–D).

### Fungal internal ROS patterns are different from those generated *in planta*

**Figure 6:**
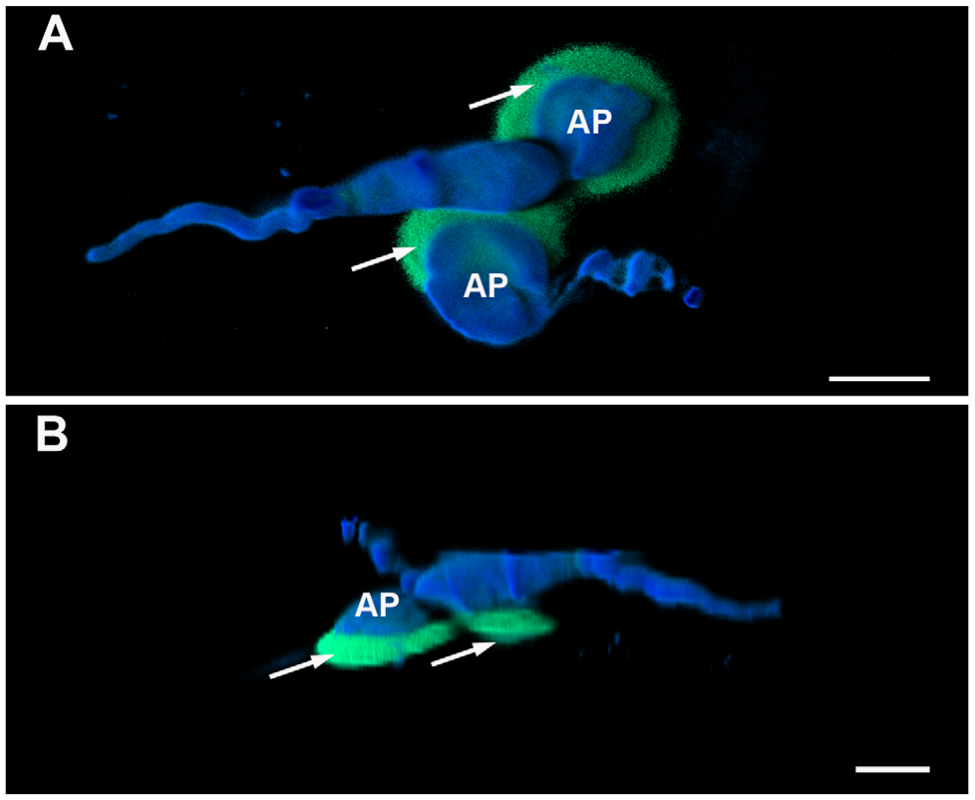
The ROS observed after inoculation with *Δhyr1* conidia as a disk-shaped halo located beneath appressoria. (**A**) A 3-D projection of confocal images with the ROS stain H_2_DCFDA showed a halo (green) of ROS around and beneath the appressoria (blue; AP), which emanated from two nearby conidia. (**B**) A side-view of panel A showed that the halo was a thin layer of ROS located beneath the appressoria. The ROS halo sits directly between the AP and the plant surface. Scale bar = 10 µm**.**

**Figure 7:**
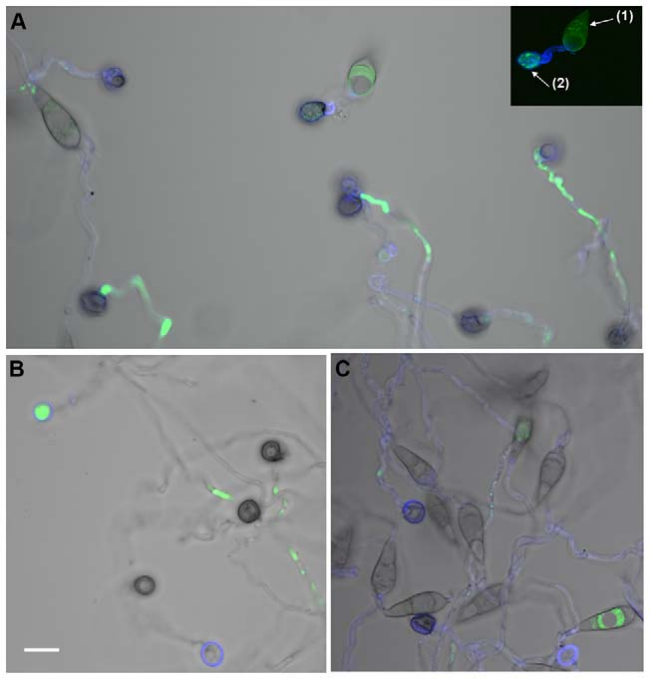
*Δhyr1* (B25) conidia on gel-bond were similar to wild type in terms of ROS production. Staining was performed 24 hpi; Calcofluor White was used to stain the cell walls (blue) and H_2_DCFDA was used to stain the ROS (green). Conidia of (**A**) *Δhyr1* (B25), (**B**) wild type (70-15) and (**C**) ectopic (B40). A transmitted light image was taken as well, and overlaid with the fluorescent image. The inset in panel A showed the fluorescence image of the conidium (1) and appressorium (2). Images were taken using confocal microscopy. Scale bar  = 10 µm.

Figure 5C suggested that reactive oxygen species localized mainly around the appressoria. Upon closer inspection, we observed that the ROS “haloes” around the appressoria usually localized directly underneath the appressoria (Figure 6). Previous studies had demonstrated that the rice blast fungus also generates internal ROS during infection-related development, particularly during appressorial maturation and furthermore, that ROS can be secreted from the fungus itself [5]. In order to identify the source of the reactive oxygen species detected in our experiment, we inoculated *M. oryzae* onto the hydrophobic side of gel-bond, which can mimic the plant surface and induce ROS production *in vitro* [32]. The result shown in Figure 7 indicated that first, *M. oryzae* does generate ROS during germ tube and appressorial formation; second, the reactive oxygen species generated by *M. oryzae* were mostly intracellular and did not appear to be secreted or defused; and finally, that ROS were relatively weak in the fungal structures by 24 hpi. These observations occurred in the wild type, ectopic and mutant lines, indicating little difference in internal ROS levels regardless of the presence of *HYR1*. Altogether, these results were different from what we observed *in planta*, which was a strong ROS signal from 24–48 hpi.

### Three lines of evidence suggest ROS is most likely plant-generated

**Figure 8:**
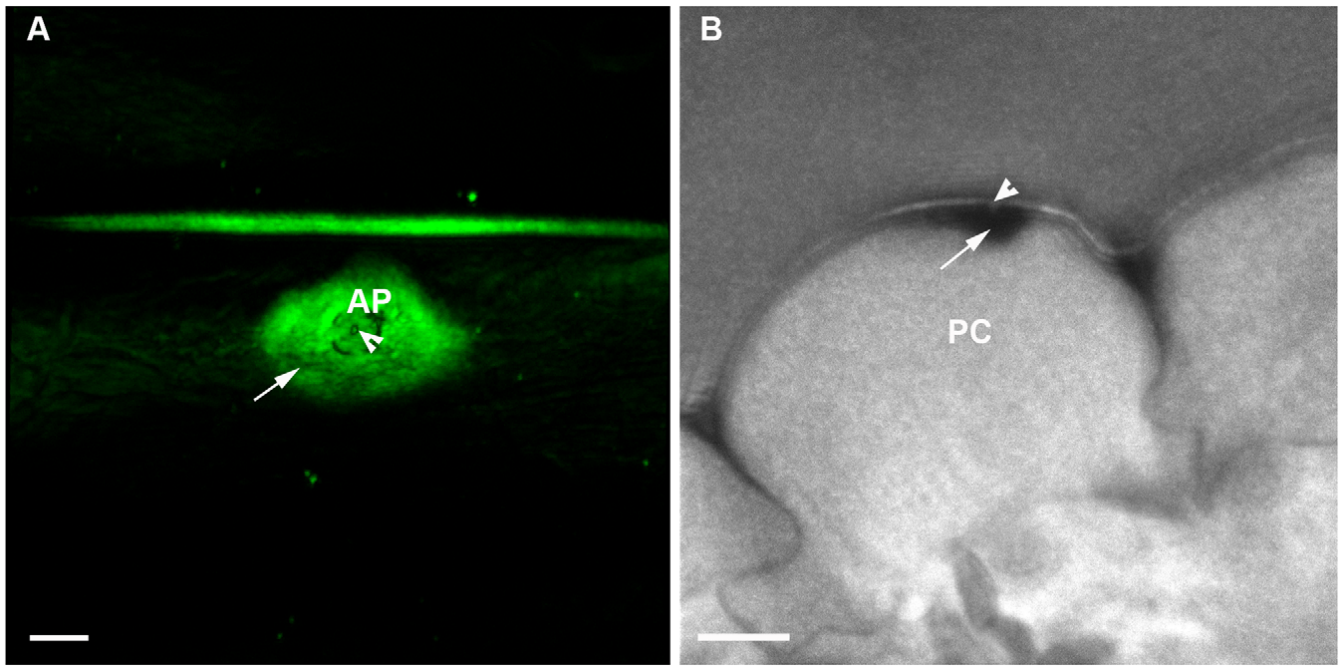
*Δhyr1* appressorial-localized ROS appeared to be plant-generated. (**A**) Reflection confocal imaging with the ROS stain DAB shows a wide ROS signal (arrow) around and beneath the appressorial attachment site (AP). In the middle of the appressorium attachment site was the putative penetration peg site (arrowhead). (**B**) The same interaction site as Fig. 8A, embedded in epoxy resin and imaged under confocal microscopy revealed DAB deposited (arrow) beneath and surrounding an attempted penetration site (arrowhead). The deposit was located up against the plant cell wall (PC) on the inside of the cell. Scale bar = 5 µm.

In order to identify the source of the ROS detected during susceptible interactions, we used diamino-benzidine (DAB) to study the ROS distribution pattern. Barley leaves were inoculated with *Δhyr1* mutant then stained with DAB and imaged using confocal reflected light signal to visualize the DAB deposits from a top view of an interaction site (Figure 8A). The leaf samples from this same interaction site was processed further and embedded in epoxy resin to obtain a cross-section using a correlative microcopy approach. The confocal images suggested that the dark region (DAB) was localized immediately adjacent and inside the plant cell wall (Figure 8B) centered around the penetration peg (arrowhead - Figure 8B).

**Figure 9:**
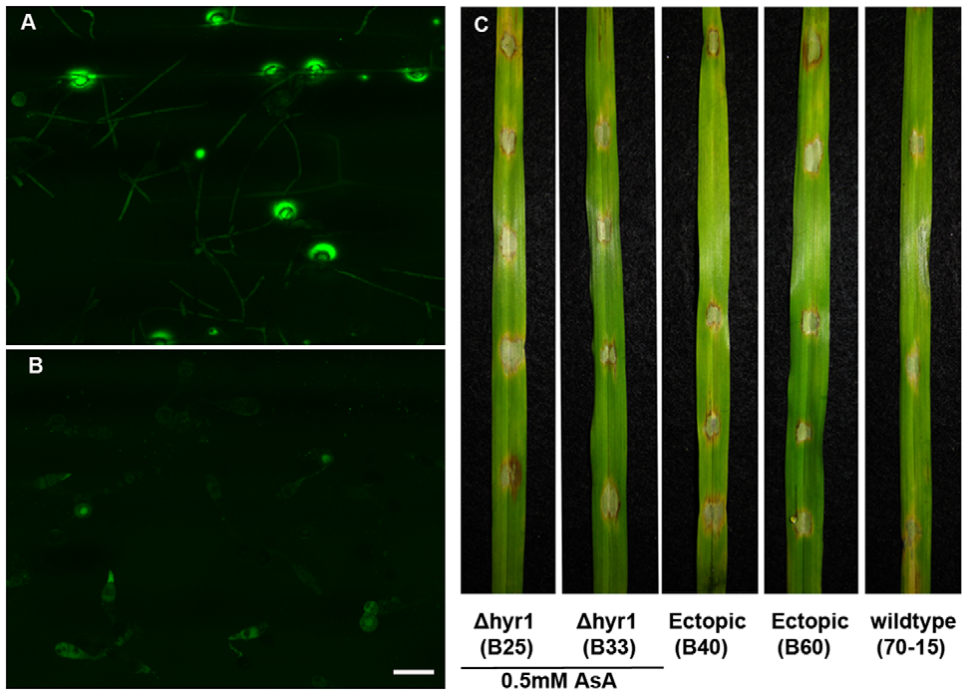
ROS scavenging in the plant rescued the *hyr1* mutant phenotype. (**A**) Conidia of *Δhyr1 (B25)* were mixed with 0.5 mM ascorbic acid and inoculated onto the leaf surface. Infected leaves were stained for ROS 24 hpi. (**B**) Conidia were mixed with water and inoculated onto the leaf surface. Leaves were first treated with 0.5 mM ascorbic acid for 1 hour and stained for ROS 24 hpi. (**C**) From left to right: *Δhyr1 (B25), Δhyr1 (B33)* (where susceptible barley leaves were treated with 0.5 mM ascorbic acid for 1 hour and then inoculated with mutant spores in water) ectopic (B40), ectopic (B60), wild type (70-15). Scale bar  = 20 µm for all confocal images.

The second piece of evidence resulted from scavenging for ROS with ascorbic acid, an antioxidant that detoxifies hydrogen peroxide [33]. When 0.5 mM ascorbic acid was mixed with *Δhyr1* mutant conidia, inoculated onto plants and stained with H_2_DCFDA, ROS haloes were clearly observed (Figure 9A). However, when barley leaves were pre-treated with ascorbic acid, then inoculated and stained with H_2_DCFDA, almost no ROS haloes were detected (Figure 9B). This experiment was repeated with another ROS-inhibitor called DPI (diphehyleneiodonium chloride), with similar results (data not shown). Ascorbic acid-treated leaves were also inoculated with mutant conidia and allowed to incubate in the growth chamber for six days, after which time we observed wild type lesions (Figure 9C). This suggested that the ROS haloes observed in this experiment are likely originated from the plant.

Futhermore, we analyzed previously characterized *nox1* and *nox2* mutants for ROS haloes; in *M. oryzae*, *NOX1* and *NOX2* code for NAPDH oxidases, and are largely responsible for producing internal ROS [5]. We hypothesized that if ROS was emanating from the plant, than the loss of the *NOX* genes should have no effect on haloes. Overall, haloes can still be produced when either of the *nox* mutants, or its parental strain, Guy11 was inoculated onto barley leaves (Figure S4A–F). While there was a slight significant difference among the number of haloes observed when looking at the individual mutants (*nox1* made slightly more than *nox2*), there was no significant difference between mutants and wild type (20–30 appressoria were counted per strain, and the percentage of those with haloes, reported; Figure S4G).

### MoHyr1 has an effect on later, but not immediate, plant-produced ROS

Since our data strongly suggested that *Δhyr1* mutants had a lower capacity to eradicate plant-generated ROS during early stages of infection. Our next goal was to determine whether this gene played a role in fungal tolerance to ROS generated immediately following inoculation. In order to carry out this experiment, we inoculated susceptible barley leaves with either the *Δhyr1* mutants or the wild type conidia, and imaged them 1 hpi. The ROS dye H_2_DCFDA was injected directly into the leaves, so the result only showed the redox status inside the leaves, and not inside the fungus, which might have skewed the results. Our data revealed that ROS was detected 1 hpi, which indicated that the plant detected and responded to the pathogen at an early time point (indicated by ROS fluorescence in the mesophyll cells; Figure S5A). A quantitative analysis of the signal intensities by ImageJ (available at http://rsb.info.nih.gov/ij; developed by Wayne Rasband, National Institutes of Health, Bethesda, MD) revealed no significant differences when inoculated with the *Δhyr1* mutants or with the wild type conidia (Figure S5B). We thus concluded that the *MoHYR1* gene does not play a role in ameliorating an early, or immediate, plant defense response.

**Figure 10:**
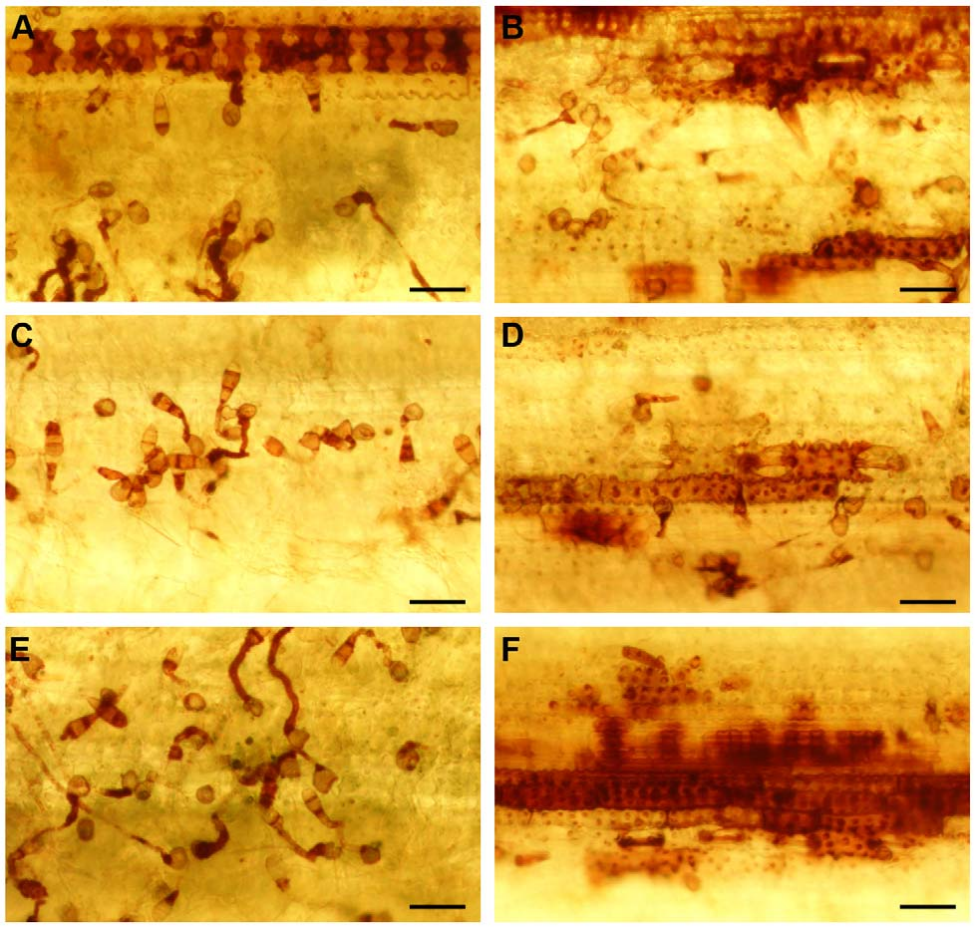
Mutants have more DAB staining than wild type revealed a stronger plant reaction. DAB staining was performed on wild type (70-15) conidia (**A, C, E**) and *Δhyr1* (B25) mutant conidia (**B, D, F**) 24hpi. Wild type (70-15) conidia on the leaf surface shows DAB staining mostly the fungal structures while *Δhyr1* (B25) mutant conidia elicit a stronger ROS plant reaction. Images were generated with a transmitted light microscope. Scale bars  = 100 µm.

To test whether *MoHYR1* had any impact on plant-produced ROS that may occur later during infection, we inoculated *Δhyr1* mutant conidia or wild type conidia onto barley leaves and stained with DAB at 24 hpi (Figure 10). Results indicated that the *Δhyr1* mutant was unable to block ROS produced at 24 hpi, where the ROS was both detected in an entire plant epidermal cell, as well as in plant cells that were not in direct contact with the pathogen (Figure 10).

### ROS generated during the infection process are related to cell wall appositions (CWAs)

**Figure 11:**
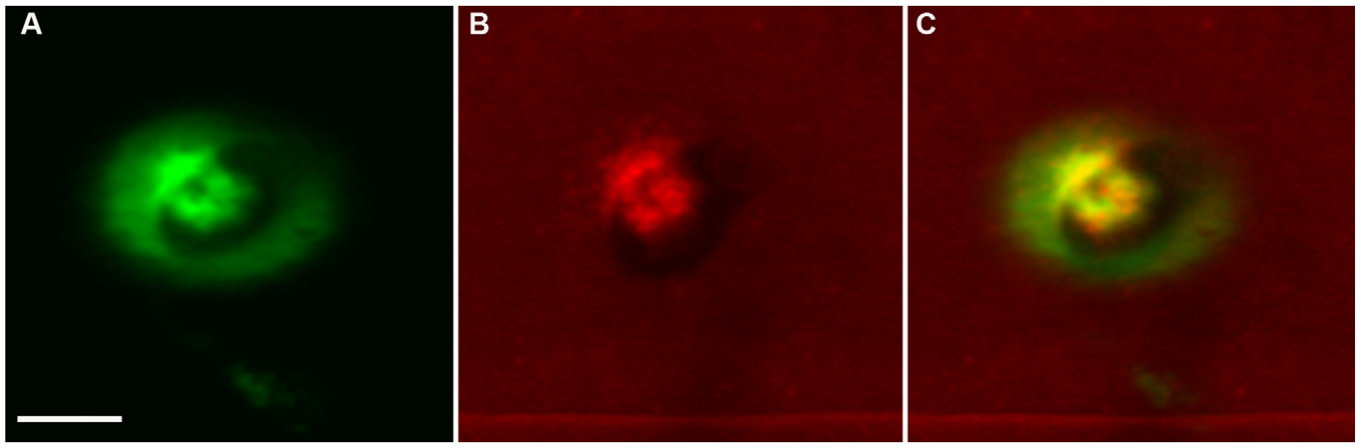
Two plant defense responses overlap when the *Δhyr1* mutant conidia were inoculated onto leaves. Correlative images show plant reaction underneath appressoria 24 hpi. (A) ROS staining; (B) aniline blue staining; (C) merged image of panels A and B. Images were processed sequentially (ROS followed by aniline blue), imaged by confocal microscopy and correlated. Scale bar = 2.5 µm.

It has been documented that the presence of reactive oxygen species around CWAs is a biochemical marker for non-penetrated cells during the interaction between barley and barley powdery mildew, *Blumeria graminis* [34]. To determine whether the ROS observed during a susceptible barley-*M. oryzae* was related to CWAs, we performed aniline blue staining on inoculated leaves. At 24 hpi, we found callose deposits specifically localized around the appressoria and penetration sites (Figure 11). Sequential correlative staining with H_2_DCFDA for ROS followed by analine blue for callose, showed a strong positional correlation between the two host responses when overlaid (Figure 11C).

**Figure 12:**
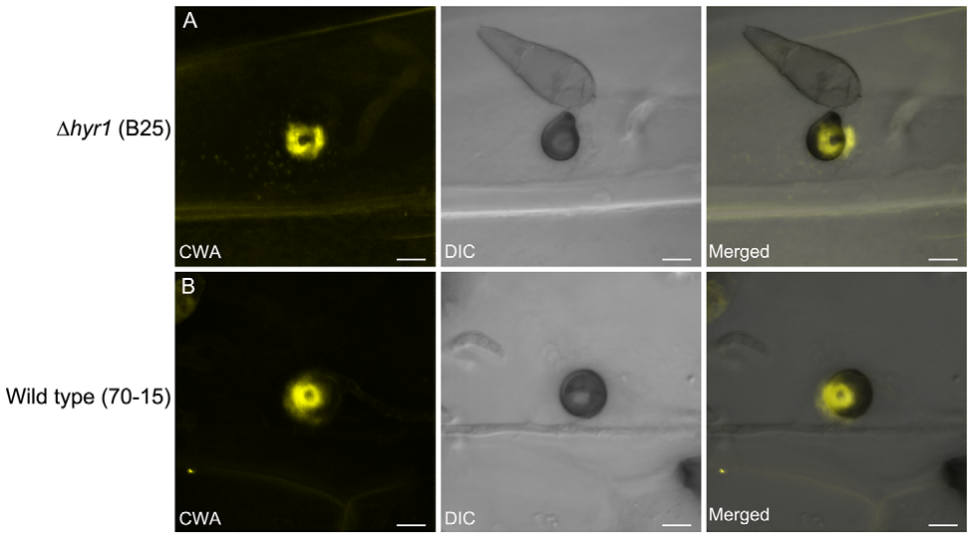
Putative plant-generated cell wall appositions surround the penetration sites 40 hpi. Confocal 3-D maximum intensity projections of aniline blue stained infected leaves showed cell wall appositions. (**A**) A representative cell wall apposition (yellow) shown here was detected in barley 40 hpi with *Δhyr1* (B25) mutant conidia. (**B**) Comparable cell wall appositions (yellow) were also detected in barley 40 hpi after inoculation with wild type (70-15). Transmitted light images were merged with 3-D confocal data to aid in visualization of plant and fungal structures. Scale bars = 5 µm.

CWAs are believed to physically block pathogen penetration [34]. To further characterize the CWAs formed during the barley- *M. oryzae* interaction, we examined leaves that had been inoculated with *M. oryzae* 24 and 40 hpi with either mutant or wild type conidia. The result showed that classical CWAs were formed within 24 hpi in both strains and no other differences in CWA morphology could be detected (Figure 12).

### *MoHYR1* regulates other ROS-related genes in *M. oryzae*

**Figure 13:**
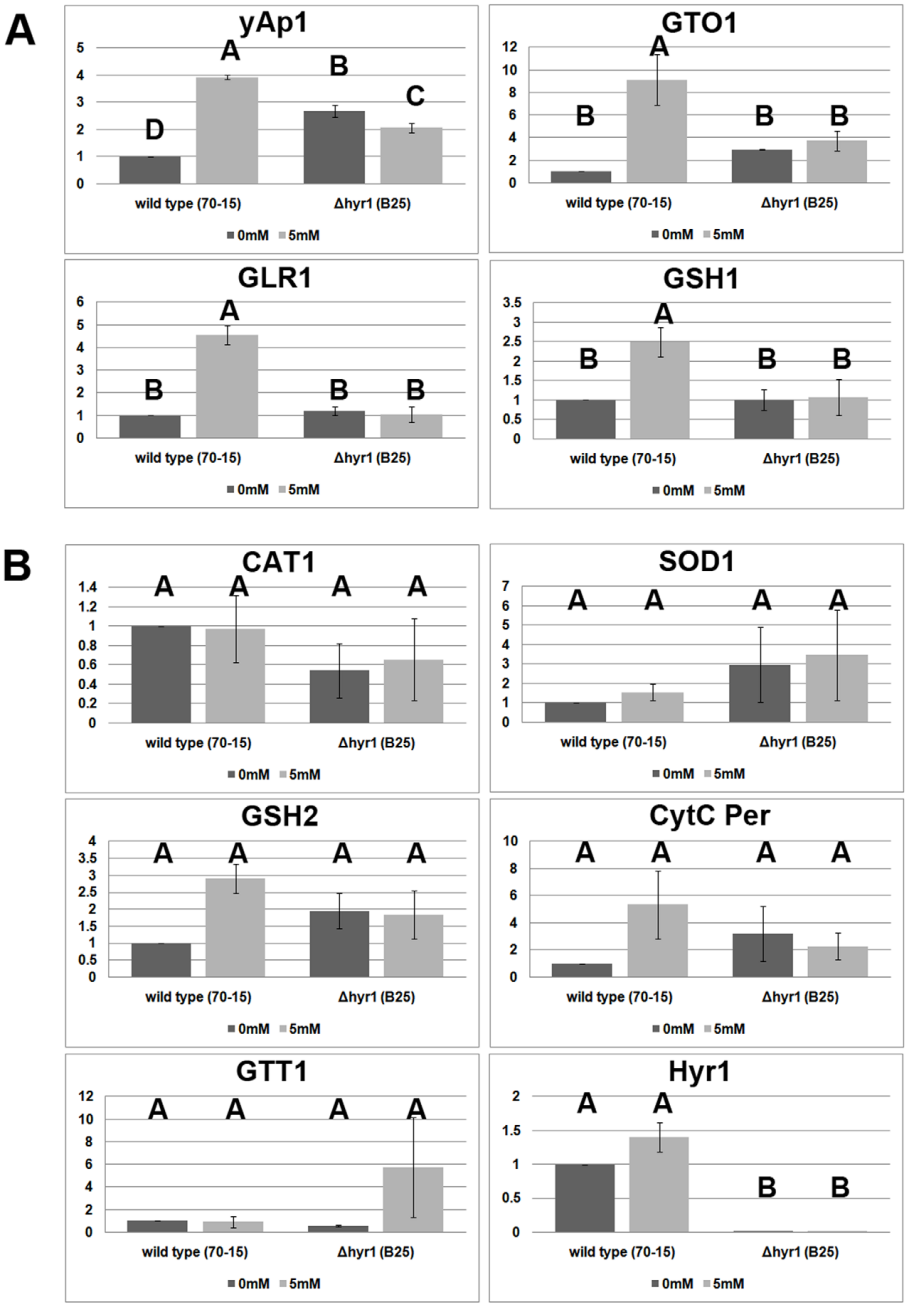
Antioxidant gene orthologs have altered expression in the *Δhyr1* mutant versus wild type. Wild type (70-15) and *Δhyr1* mutant (B25) were grown in 0 mM and 5 mM hydrogen peroxide and collected 1 hour after immersion. RNA was extracted and real-time qRT-PCR performed on three biological replicates. (**A**) The *YAP1*, *GTO1*, *GLR1* and *GSH1* all increase in expression in wild type upon H_2_O_2_ challenge, but the latter three display low levels in the mutant. (**B**) *CAT1*, *SOD1*, *GSH2*, *GTT1* and cyt *c* peroxidase do not display significant changes in expression. *MoHYR1* expression is abolished in the mutants. Letters over bars represent statistically significant differences between expression changes of the genes (statistics were generated using student t-test with p-value <0.05).

Given the fact that increased ROS accumulation occurs in the absence of MoHYR1, we next tried to determine whether the ROS scavenging system was impaired in the *Δhyr1* mutants. We used real-time quantitative real time reverse transcription PCR (real-time qRT-PCR) to compare the expression of general antioxidant and redox control gene orthologs in both *M. oryzae* wild type and *Δhyr1* strains (Figure 13). Primer pairs for the following genes were employed to examine gene expression: *YAP1* (MGG_12814.6), *GSH1* (γ-glutamylcysteine synthetase; MGG_07317.6), *GSH2* (glutathione synthetase; MGG_06454.6), *GLR1* (glutathione reductase; MGG_12749.6), *GTT1* (glutathione transferase 1; MGG_05677.6), *SOD1* (Cu/Zn superoxide dismutase; MGG_03350.6), *CAT1* (catalase 1; MGG_10061.6), *GTO1* (omega class glutathione transferase 1; MGG_05367.6), and cyt *c* per (cytochrome *c* peroxidase; MGG_10368.6). The housekeeping gene encoding *Ubc* (ubiquitin conjugating enzyme; MGG_04081.6) was used as an internal control. We also included the gene MoHYR1 (MGG_07460.6) in this experiment to confirm its deletion in the mutant lines. The expression patterns of these ten genes were placed into two categories. The first category (Figure 13A) is comprised of four genes that show increased expression in the wild type strain after induction with hydrogen peroxide, while expression in the mutant line is low and unchanging. *GTT1*, *GR* and *GSH1* belong to this category, along with the *HYR1* partner protein *YAP1*; *YAP1* also shows slight but significant differences in expression in the *Δhyr1* mutant line with and without H_2_O_2_, and has a higher expression level compared to the wild type strain without ROS. The second category contains genes whose expression does not significantly change, both in response to H_2_O_2_, as well as in the presence of the *MoHYR1* gene. This category includes six genes: *cyt c per*, *CAT I*, *Cu/Zn SOD*, *GTT I*, *GSHII* and *MoHYR1* (Figure 13B). *HYR1* shows no expression at all in the mutant line, which was to be expected.

### Hyr1 cellular localization

**Figure 14:**
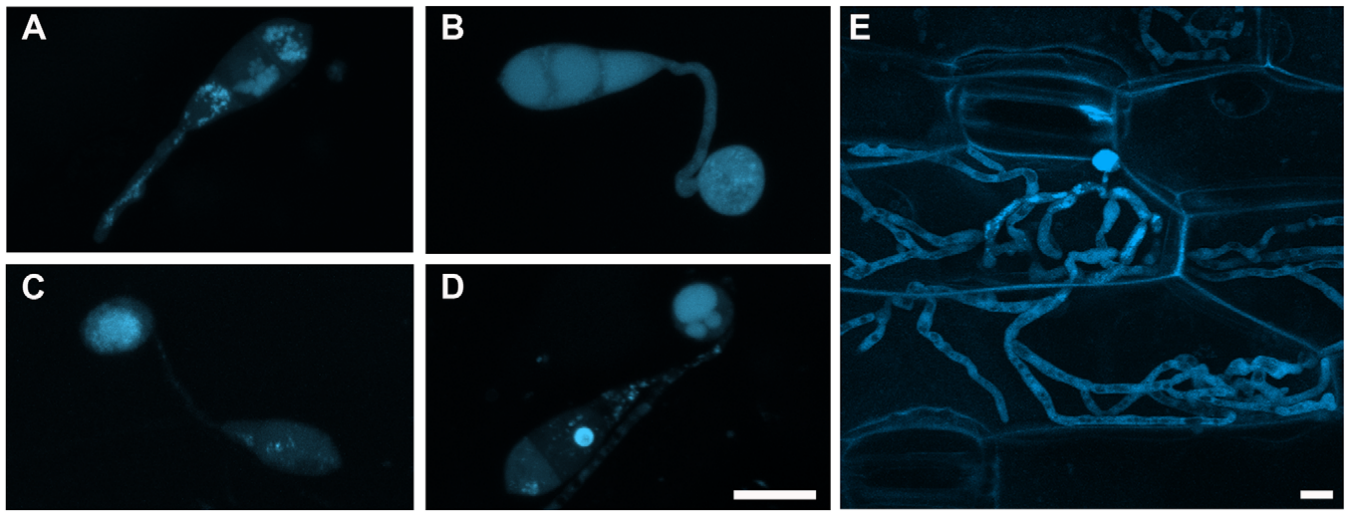
MoHYR1 changed localization during pre-penetration events on the surface of a leaf. The MoHYR1 coding sequence was fused to the cerulean fluorescent protein to study protein localization during early infection. (**A**) HYR1 at 1 hpi with putative vacuole location and low level cytoplasmic distribution; the germ tubes has formed, but no appressorium. (**B**) HYR1 at 6 hpi with increased cytoplasmic localization where it is likely to be required to function in ROS scavenging; an immature appressorium was apparent. (**C**) HYR1 at 12 hpi with cytoplasmic location; a mature appressorium was apparent. (**D**) HYR1 at 24 hpi with vacuole and low level cytoplasmic localization in the appressorium. (**E**) HYR1 at 72 hpi again showing cytoplasmic localization. Images were taken with confocal microscopy and all experiments were done on the surface of barley leaves. Scale bar shown  = 10 µm for all images.

We evaluated the sub-cellular localization pattern of the MoHYR1 protein during infection, conidia of a *M. oryzae* deletion line (*Δhyr1* B33) transformed with cerulean-MoHYR1 N-terminal fusion (the same construct that was used for complementation), was inoculated onto barley leaves and observed during the following time points: 1 hpi, 6 hpi, 12 hpi, 24 hpi and 72hpi. At 1 hpi, MoHYR1 was mainly localized in the conidial vacuoles and with low levels in the cytoplasm. When the germ tube formed, the protein was present throughout the germ tube (Figure 14A). At 6 hpi, the MoHYR1 protein showed increased cytoplasmic localization in the appressorium and conidium and at 12 hours, a concentration of HYR1 in the appressorial cytoplasm (Figures 14B and C). At the later time point, 24 hpi, the protein appeared to be localized in the vacuoles with reduced levels in the cytoplasm (Figure 14D), and a later, invasive stage time point suggests the protein was again cytoplasmically localized (Figure 14E).

## Discussion

During the interaction between the pathogens and plants, plants mount defense mechanisms to protect themselves from pathogens. The cellular environment within the host can represent a major source of stress towards the invaders [16]. Pathogens, on the other hand, must possess adaptive mechanisms in order to survive. In this study, we hypothesized that the *M. oryzae* HYR1 protein defines one such mechanism, the glutathione synthesis pathway, involved in coping with the oxidative environment generated by plant defenses.

### MoHYR1 is necessary for ROS detoxification and full virulence

In *M. oryzae, MoHYR1* is the only sequence homolog of the yeast glutathione-dependent peroxidase, *HYR1p*, formerly termed *Gpx3* [35]. In yeast, *HYR1p* senses H_2_O_2_ through two highly conserved cysteines that are redox sensitive. Mutations in either of these two cysteines leads to a non-functional HYR1 [18]. Indeed, we found that the wild type *MoHYR1*, but not the *MoHYR1* cysteine mutants, was able to partially rescue the yeast *HYR1p* mutant on non-permissive levels of H_2_O_2_. This result is similar to *Δyap1* yeast mutants complemented with homologs from two pathogenic filamentous fungi, *Cochliobolus heterostrophus* and *Ustilago maydis*, as both homologs partially complemented the yeast mutation [20], [23]. These data suggested that *MoHYR1* may function similarly during redox sensing and the subsequent signaling that leads to ROS detoxification. This model was further supported by the presence of ROS haloes located underneath appressoria during infection with a much greater frequency in the *Δhyr1* mutant compared to the wild type strain.

The increase in ROS haloes in *Δhyr1* mutants correlated with significantly smaller lesions sizes when inoculated on susceptible rice and barley plants, suggesting that ROS scavenging regulated by MoHYR1 was required for full virulence. This was supported by a rescuing of the *Δhyr1* mutant phenotype to wild type lesions by scavenging plant-derived ROS with ascorbic acid or disrupting plant-derived ROS generation with DPI. These results were similar to a gene recently reported on in the rice blast fungus called *DES1* for Defense Suppressor 1 [14]. *DES1* was also involved in virulence and triggers a stronger plant response upon infection, manifested by both an increase of the oxidative burst, as well as expression of two plant defense genes. Intriguingly, *DES1* has no known functional domains and from sequence analysis, its function cannot be predicted, although it is well-conserved throughout fungi. It is also noteworthy that expression of *MoHYR1* was tested in the *Δdes1* mutant, and found to be slightly down-regulated. This could suggest that *HYR1* and *DES1* represent two semi-redundant, semi-dependent mechanisms evolved to cope with the plant defense response. Equally interesting is a gene recently identified in the plant and human fungal pathogens, *Alternaria brassicicola* and *Aspergillus fumigatus*, respectively, called *tmpL* [16]. This membrane-localized gene contains a FAD/NADP-binding domain and had not yet been studied in fungi. A deletion of *tmpL* resulted in a severely reduced virulence defect and hypersensitivity of exogenous oxidative stresses, however when the *YAP1* gene was over-expressed in the deletion line, it rescued these and other mutant phenotypes, suggesting *tmpL*, *YAP1* and presumably *HYR1* may act in a concerted pathway to sense and trigger ROS scavenging pathways.

### MoHYR1 helps the fungus negotiate a hostile host environment

A successful pathogen, which has the ability to detoxify ROS, will subsequently have fewer barriers to overcome before reaching its ultimate goal, which are the cell contents. Our results with the *MoHYR1* gene suggest that while there might be no effect of *MoHYR1* on ROS that's produced immediately by the plant (Figure S3), there is subsequent ROS production which *MoHYR1* clearly helps the fungus overcome (Figure 10). Metabolic profiling performed by Talbot and colleagues (2008) provides support for this concept, revealing a *M. oryzae*-induced host metabolism re-programming that suppressed or delayed plant-produced ROS during susceptible interactions.

Although supporting evidence has shown that *M. oryzae* can produce ROS during infection related development [5], through scavenging experiments, the ROS observed in our studies appear to be largely plant-generated. Internal fungal ROS was unaffected by the absence of the *MoHYR1* gene *in vitro.* Furthermore, ROS haloes were not disrupted by the ROS scavenger, ascorbic acid, when applied only to conidia, but were disrupted when ascorbic acid was specifically applied to leaves. Several pathways for plant-generated ROS include cell wall-bound peroxidases [1]. Plants defend themselves against pathogens by a battery of cell wall-associated defense reactions, including generation of ROS and cross-linking of lignin compounds [34]. During the interaction between a French bean (*Phaseolus vulgaris*) and a cell wall elicitor from *Colletotrichum lindemuthianum*, ROS appears to originate from cell wall peroxidases [36]. Apoplastic alkalization has been shown to be important in this process [34]. ROS generated from cell wall peroxidases also serve as key molecules required for lignification and cross-linking of cell walls [34]. In a study carried out between barley and the powdery mildew fungus, barley cell wall localized peroxidase *HvRBOHA* is responsible for generating H_2_O_2_, which was only present in non-penetrated cells [37]. Our results, particularly in Figure 8B, suggest ROS localized up against the plant cell wall. Further investigations into *M. oryzae*-host interactions will include analyzing plant defense-related genes, including the barley cell wall peroxidase.

Callose and ROS are two plant defensive compounds known to be involved in cell wall appositions, which are deposited during both compatible and incompatible interactions [34]. H_2_O_2_ played an important role in this process and enzymatic removal of H_2_O_2_ by catalase significantly reduces the frequency of phenolic deposition [34]. Several components were reported to be essential for this oxidative burst: peroxidases, a calcium influx and K^+^ Cl^−^ efflux, extracellular alkalization, and post-Golgi vesicles [38]. ROS around the CWA areas might function as signal compounds to gather the vesicles and components needed for mature CWAs. We observed that ROS and callose deposits were positionally related during attempted penetration by both wild type and *Δhyr1* mutants, immediately below the appressorium. From this result, we hypothesize that ROS generated by plant defenses activates CWA formation in a susceptible host and experiments to determine the timing of deposition of ROS versus callose are currently underway.

A hypothesis that follows from these data is that when the *MoHYR1* gene is deleted, the plant responds as though it's being challenged with an avirulent pathogen. As early as 12 hours post inoculation, we observed that barley leaves inoculated with *Δhyr1* mutants showed higher ROS signals compared with leaves inoculated with wild type. These data were consistent using two staining methods, H_2_DCFDA and DAB. In leaves inoculated with wild type, ROS was detected around appressoria but was mostly observed inside fungal structures. However, ROS was seen both around appressoria and adjacent cells when inoculated with the *Δhyr1* mutants. Whole cells filled with ROS were also observed when inoculated with *Δhyr1* mutants, which was related with HR-type cell death. All these data indicated that *HYR1* might function to suppress later plant-generated ROS, either by detoxifying it directly, or manipulating plant ROS secretion-related gene expression.

### MoHYR1 regulates several genes involved in ROS-scavenging

While our data showed that HYR1 likely played an important role in ROS-detoxification processes, our experiments did not preclude other ROS tolerance mechanisms in the fungus, particularly since mutants were reduced in virulence, but not completely non-pathogenic. Such mechanisms might involve the aforementioned *DES1* and *tmpL* genes. Currently, we are characterizing the *MoYAP1* homolog in *M. oryzae*; our initial *Δyap1* mutant data suggested this gene was dispensable for pathogenicity, much like what has been found in *Botrytis cinerea*, *Aspergillus fumigatus* and *Cochliobolus heterostrophus* [23], [25], [26]. Intriguingly, *YAP1* did appear to be essential for virulence in *Ustilago maydis* and *Alternaria alternata* [20], [25], suggesting that fungal lifestyle (i.e. necrotrophic vs. biotroph) had little to do with this particular oxidative stress pathway, and further supporting redundant pathways. Our real-time qRT-PCR data showed that *YAP1* increases in expression when wild type was challenged with H_2_O_2_ and we also noted a decrease in *YAP1* gene expression in the *Δhyr1* mutant background. One interpretation of this result was that the fungal cell might be compensating for the absence of *HYR1*, by boosting expression of its partner gene.

The glutathione pathway-related genes *GLR1*, *GTO1* and *GSH1*, all increased during H_2_O_2_ challenge in the wild type however had extremely decreased expression in the mutant line, regardless of ROS. This suggested that these genes were reliant upon *HYR1*, which was not unexpected, since the glutathione pathway was shown to be regulated *YAP1*, which occurs after interacting with HYR1 [17]. Our results were also in keeping with the *C. heterostropus Yap1* homolog mutant *Δchap1*, which showed extremely low levels of both *GLR1* and *GSH1* [23]. Interestingly, we did not observe any of the other genes increasing in expression in the mutant background; this suggested that at least for the genes that we chose such as *CAT1* and *SOD1*, they did not provide compensatory mechanisms for a loss of *HYR1*. While this is one hypothesis, it is also possible that these genes are regulated at the protein level, as was found in the *A. fumigatus* mutant, *ΔAfyap1*; both *CAT1* and *SOD1* were among the proteins down-regulated in the mutant [39], and this could also hold true for the *Δhyr1* mutant. Likewise, catalase, SOD and peroxidase activities were measured in the *A. alternata* mutant *ΔAaAp1* [25]. A transcriptomic study on the *Δhyr1* deletion mutant would answer many of these questions; further, such a study would uncover redundant pathways of ROS detoxification masked by the presence of MoHYR1.

### Localization of the MoHYR1 protein

While numerous studies have examined localization of the Yap1p, we were unable to find any studies on the localization of HYR1 either in yeast or filamentous fungi. Our data revealed that the HYR1 protein mostly localized either to the cytosol or to vacuoles, during early stage infection events on barley (germ tube, early appressorial formation, appressorial maturation and penetration). At one hpi, MoHYR1 was mainly moving through the germ tube, although it was difficult to definitively ascertain which organelle it might be associated with. At twelve hpi, the MoHYR1 protein shows cytoplasmic localization, mainly expressed in the cytosol of the appressorium. We suspect that by twenty-four hours, the fungus had penetrated and gained ingress to the first epidermal cell; indeed cell biology studies on events following initial penetration suggested that *M. oryzae* bulbous hyphae fill an entire rice leaf sheath cell and were in the process of moving onto the next one by twenty-seven hours post-inoculation [40]. Its vacuolar localization at this time-point could reflect that fact that it was no longer needed by the fungus, which had circumvented the plant's oxidative burst and at that point growing in the first epidermal cell. We examined a later time-point at 72 hpi and found the HYR1 gene to be once again cytoplasmically localized, perhaps indicating a requirement for this pathway at the invasive growth stage.

### Conclusions and future directions

In conclusion, we identified and characterized the *MoHYR1* gene, a functional homolog of the yeast *Hyr1* (or *Gpx3*) gene. Although MoHYR1 does not cause dramatic effects in the disease phenotype, it nevertheless played an important role in virulence. This effect appeared to be related to the deletion mutant's inability to tolerate plant-generated ROS, or at least to do so in a timely and effective manner to cause wild type levels of disease. Together, our results help to define a mechanism that, while well-studied in yeast, has not yet been examined in filamentous fungi; furthermore, our studies pose additional questions to be answered regarding the role of the glutathione pathway in scavenging ROS in filamentous fungi, how this aids in pathogenicity and what other underlying redundant scavenging pathways exist.

## Materials and Methods

### *M. oryzae* strains and growth conditions

Rice-infecting *M. oryzae*, strain 70–15 (Fungal Genetics Stock Center 8958) was used as the wild type strain throughout this project, and the strain from which mutants and transgenics were derived. All strains were maintained at 25°C under constant fluorescent light on complete medium (CM 1 liter: 10 g sucrose, 6 g yeast extract, 6 g casamino acid, 1 ml trace element). Oatmeal agar medium (OAM 1 liter: 50 g oatmeal and 15 g agar) was used for sporulation. Conidia were harvested 10–12 days after plating.

### Yeast strains and complementation assays

Yeast strains BY4741 (wild type) and BY4741 YIR037W (Δhyr1 mutant) were ordered from the American Type Culture Collection, grown out and maintained on YPD medium. Constructs for transformation were built using standard PCR reaction conditions and programs; briefly, pJS371 used overlapping primers to make an intron-free version of the MoHYR1 gene in pJS318. Using the intron-free plasmid, overlapping primers were used to make Cys39Ala and Cys88Ala mutant versions of the coding sequence. These were cloned into pCRScript (pJS372 & pJS373, respectively). The yeast HYR1 gene (ScHYR1) was then amplified from Sc46 and cloned into pRS423, the His3 episomal plasmid, pJS374. These plasmids then form the basis of the genes to be tested: MoHYR1 wild type, the 2 cysteine mutants of MoHYR1 and the ScHYR1 gene. These four genes are under the same promoter and terminator. Therefore ScHYR1 was engineered to have an NcoI site at the ATG and a BamHI site at the beginning of the terminator (pJS375). Since the Magnaporthe gene has a natural NcoI site at the ATG, the 3 genes of the MoHYR1 are cloned into pJS379 as NcoI/BamHI fragments (pJS381, pJS382, pJS383).

For the complementation assays, five-microliter drops from serial dilutions from cultures with anOD600 of 0.5 were spotted on plates with and without 0, 2 and 4 mM H2O2 and grown for 2 days at 30°C. This experiment was repeated 10 times. In total, the following plasmids were used in this part of the study:

pSM387 ( = pRS423) HIS3 yeast episomal plasmid; pJS374 pSM387 + ScHYR1;

pJS381 ScHYR1-Pro::MoHYR1::ScHYR1Term;

pJS382 ScHYR1-Pro::MoHYR1_Cys36Ala::ScHYR1Term;

pJS383 ScHYR1-Pro::MoHYR1_Cys82Ala::ScHYR1Term.

### Plants cultivars and growth conditions

Rice cultivar Maratelli (a gift from the Dean Lab; Raleigh, NC) and barley cultivar Lacey (Johnny's Selected Seeds; Winslow, ME) were used throughout this study, as both are susceptible to *M. oryzae* strain 70–15. Rice was grown in a growth chamber at 80% humidity, and 12 h:12 h day:night cycles, at 28°C. Barley was grown in a growth chamber at 60% humidity, and 12 h:12 h day:night cycles, at 24°C (day) and 22°C (night).

### Targeted deletion of Hyr1

The targeted gene deletion was accomplished using the homologous recombination method. We amplified 5′ and 3′ flanking regions of *Hyr1* using primer pairs #1 and 2 (Table S2). Flanking regions were then linked via adaptor-mediated PCR to a 1.3 kb *HPH* coding sequence, providing resistance to the antibiotic hygromycin (Alexis Biochemicals, San Diego, CA). The entire length of the deletion fragment was 3.7 kb. Fungal protoplasts of the wild type 70-15 were directly transformed with the nested PCR product (primers used were forward primer of primer pair #1 and reverse primer of primer pair #2). Protoplast generation and subsequent transformation were conducted by following established procedures [41]. To confirm the knockout mutant, the genomic DNA of candidate strains was extracted and amplified with primer pairs #3, 4 and 5 (Table S2).

### *In vitro* H_2_O_2_ growth assessment of *Δhyr1* mutants

Equal-sized pieces of mycelia were cut with #3 cork-borer tool (0.7 cm in diameter), and immersed in 10 ml of liquid CM at 25°C in darkness. Colonies were grown in CM containing H_2_O_2_ at concentrations of 0 mM, 5 mM and 10 mM. Colonies were removed from each well, vacuum filtered to dryness, and measured on a scale one week post-immersion.

### Pathogenicity assays

For point or drop inoculations, conidia were harvested from 12-day-old cultures grown on OMA in 20 µl of a 0.2% gelatin (Acros organics, New Jersey) suspension, for a final concentration of 1–5×10^5^ conidia/ml. Point two percent gelatin was used as a non-inoculated control for pathogenicity assays. For drop inoculations, three week old leaves of Maratelli or Lacey were detached and laid flat in a humid chamber (90 mm Petri dish with moist filter paper). Twenty microliters of conidial suspensions, or gelatin alone, were dropped onto each leaf and kept in darkness overnight at ∼25°C. The next day, remaining water drops were wicked off and moved to a growth chamber under constant fluorescent light. For spray inoculations, conidial suspensions (10 ml; concentration as above) in 0.2% gelatin were sprayed onto three week old Maratelli or Lacey seedlings. Inoculated plants were placed in a dew chamber at 25°C for 24 hours in the dark, and then transferred into the growth chamber with a photoperiod of 16 h:8 h light:dark cycles. Disease severity was assessed seven days after inoculation.

### Quantitative real-time RT-PCR of ROS-related genes and data processing

Quantitative real time reverse transcription PCR (real-time qRT-PCR) was carried out using primer pairs for the following genes: *YAP1* (MGG_12814.6), *GSH1* (MGG_07317.6), *GSH2* (MGG_06454.6), *GLR1* (MGG_12749.6), *GTT* (MGG_06747.6), *GTO1* (MGG_05677.6), *GTT1* (MGG_09138.6), *SOD1* (MGG_03350.6), *CAT1* (MGG_10061.6) and cytochrome *c* peroxidase (MGG_10368.6). The housekeeping gene encoding ubiquitin conjugating enzyme (MGG_00604.6) was used as an internal control. We also included the gene *MoHYR1* (MGG_07460.6) to confirm its deletion in the mutant lines. Primer pairs are listed in Table S3. Seventy-five nanograms of cDNA generated from mycelium grown as per the H_2_O_2_ experiments described above (generated from the 0 mM and 5 mM H_2_O_2_ samples), was used as templates for each reaction. The mycelia were fragmented in a blender as per the protocol by Mosquera et al [42], before being inoculated into liquid complete medium. After 2–3 days, the mycelia were blended again to ensure the largest amount of actively growing fungal tips. The H_2_O_2_ experiment was performed 24 hours after the 2^nd^ blending, and RNA was extracted. PCR reaction conditions were as follows for a 25 µl reaction: 13 µl H_2_O, 10 µl 5 Prime SYBR Green Master Mix (Fisher Scientific, Waltham, MA), 0.5 µl Forward Primer (for a final concentration of 2 µM; Integrated DNA Technologies, Coralville, IA), 0.5 µl Reverse Primer (for a final concentration of 2 µM) and 1 µl template DNA. Conditions for real-time quantitative RT-PCR conditions were as follows: 95°C for 2 min; 95°C for 15 sec, 58°C for 15 sec, 68°C for 20 sec (cycle 40 times); 95°C for 15 sec; 60°C for 15 sec (melting curve); 60°C –95°C for 20 min; 95°C for 15 sec; lid temperature constant at 105°C. The 2^−ΔΔCt^ method was used for generating the data. ΔΔCt is defined as ΔCt treatment - ΔCt calibrator. cDNA from the strain 70-15 in 0 mM H_2_O_2_ was used as the calibrator for comparison of gene expression in 5 mM H_2_O_2_ in both the *Δhyr1* deletion lines as well as the wild type For both the ΔCt treatment and ΔCt calibrator, ΔCt is defined as Ct gene - Ct housekeeping-gene. For the calibrator, which is 0 µM H_2_O_2_, this value would be 2^−0^ or 1. These experiments were repeated twice with similar results.

### Cloning of *MoHYR1* and generation of fusion protein

A HYR1 N-terminal cerulean fusion construct was generated by fusion PCR. Briefly, using *M. oryzae* genomic DNA as a template, a 1 kb promoter region of *HYR1* was amplified with primers 6 and 7 (Table S2). Another set of primers, 8 and 9, were used to amplify the 2.4 kb *HYR1* open reading frame. Three resulting fragments, the 1 kb promoter fragment, the 1328 bp ORF (including 709 bp of terminator sequence) and 740 bp cerulean fluorescent protein coding sequence [43], were mixed and subjected to a second fusion PCR with primers 7 and 8. The resulting 3.1 kb PCR product was generated with *BamHI* and *NotI* restriction enzymes (New England Biolabs, Beverly, MA) and cloned into pBlueScript II SK+. The construct was fully sequenced and found to be correct, hence was co-transformed into the *M. oryzae Δhyr1* knockout mutant protoplasts to make Cerulean-HYR1 fusion transformants. Transformants with expected genetic integration events were identified by PCR using primers pairs 6 and 10 (Table S2). Properly transformed *Δhyr1* mutants were also used as the complemented lines, in Figures 3 and 4, designated as “hyr1-C”.

### Detection of ROS

Ten-fourteen day old rice and eight day old barley plants were used and collected 24 hours after being inoculated with 10–12 day old conidia (methods as described above). All staining procedures were performed with both rice and barley, however barley was best-suited for microscopy, hence all micrographs shown in this study are of barley. For experiments with 29,79-dichlorofluorescin diacetate (H_2_DCFDA) (Invitrogen, Carlsbad, CA), inoculated tissue were collected and incubated for 60 min at room temperature in 5–20 mM H_2_DCFDA dissolved in DMSO (less than 0.005% final concentration), then washed with 0.1 mM KCl, 0.1 mM CaCl_2_ (pH 6.0) and left for 60 min at 22°C before experimentation. Dye excitation was at 488 nm; emitted light was detected with a 500–550 band pass emission filter. DAB staining was carried out using the protocol developed by Thordal Christensen et al [44]. Briefly, leaves were cut at the base with a razor blade and placed in a 1 mg/mL solution of DAB for 8 h under darkness at room temperature. Leaves were decolorized by immersion in ethanol (96%) for 4 h followed by 2 hours in PBS buffer before imaging. A third method of ROS detection was employed for examining ROS internal to, or secreted from, the fungus. Nitroblue tetrazolium (Sigma-Aldrich, St. Louis) was used at 4 mg/mL (in deionized water) and the staining performed for 5 min∼30 min at room temperature prior to observation.

### ROS scavenging treatments

In order to eliminate the ROS generated by fungus, conidia of *Δhyr1 (B25)* and wild type (70-15) were mixed with 0.5 mM ascorbic acid (AsA) and inoculated onto the leaf surface. Leaves were stained for ROS at 24 hpi. In order to eliminate ROS generated from the plant, leaves were first treated with 0.5 mM ascorbic acid for 1 hour. To remove excess AsA, leaves were then washed with 0.1 mM KCl, 0.1 mM CaCl_2_ (pH 6.0) buffer three times for 5 minutes each. Finally, leaves were inoculated with conidia 1hpi and stained for ROS 24 hpi. Additionally, barley leaves were injected with 5 µM DPI (diphenyleneiodonium; Sigma, St Louis), then washed and inoculated, as above.

### Detection of fungal cell wall

Calcofluor White M2R (Fluorescent brightener 28, F-6258, Sigma, St Louis) was used for detection of the fungal cell wall. We made 10,000-fold dilutions from a saturated Calcofluor White stock solution. For experiments involving conidia on gel-bond (VWR, Arlington Heights, IL), Calcofluor White was applied 1, 4, 8, 12, and 24 hours post inoculation, incubated for 15 minutes, then gently rinsed off with 1X PBS buffer. For experiments involving inoculated plants, inoculated or non-inoculated (control) leaf tissue was collected and immersed in working solution for 15 minutes, then gently rinsed with 0.1 mM KCl, 0.1 mM CaCl_2_ (pH 6.0).

### Detection of Cell Wall Appositions (CWAs)

For CWAs staining, we cleared inoculated or non-inoculated (control) leaves in ethanol:acetic acid (6:1 v/v) overnight and washed them with water. Subsequently, cleared leaves were incubated in 0.05% aniline blue (w/v) in 0.067 M K_2_HP0_4_ buffer at pH 9.2 overnight and rinsed gently in sterilized deionized water for microscopy.

### Localization of DAB

Inoculated barley leaves were stained using DAB and rinsed several times in PBS. Thereafter, samples were fixed in 2% paraformaldehyde (Electron Microscopy Sciences, Hatfield, PA) and 2% glutaraldehyde (Electron Microscopy Sciences, Hatfield, PA) in sodium cacodylate (Electron Microscopy Sciences, Hatfield, PA) buffer for 1 hour overnight. Samples were then rinsed three times, 15 min each, in sodium cacodylate and post-fixed with 2% OsO_4_ in sodium cacodylate for 3–5 hours on a rotator. Again, samples were then rinsed three times for 15 min each, with water on a rotator. Samples then underwent an ethanol dehydration series (25%, 50%, 80% ETOH; 20 min each) on a rotator. Samples were primed with 1% gamma-glycidoxylpropyl trimethoxysilane in 80% ETOH overnight at room temperature and then washed three times for 15 min each in 100% ETOH on a rotator. Samples then underwent a series of infiltrations on a rotator as follows: 100% ETOH/n-BGE (Electron Microscopy Sciences, Hatfield, PA) (1:1) for 30 min, 100% n-BGE for 30 min, n-BGE/Quetol-651 (Electron Microscopy Sciences, Hatfield, PA) (1:3) for 1 hour, n-BGE/Quetol-651 (1:1) for 1 hour, n-BGE/Quetol-651 (3:1) for 1 hour, 100% Quetol-651 for 1 hour, 100% Quetol-651 for 1 hour, 100% Quetol-651 overnight and 100% Quetol-651 for 1 hour. Finally, samples were embedded and polymerized in an oven at 60°C for about 24 hours.

### Bioinformatic and statistical analyses

BlastP analysis was done against the fully sequenced genomic database of *M. oryzae* housed at the Broad Institute, using an e-value of 1e-3. ClustalW (X2) was used to perform the full alignment and generate the phylogenetic tree. The final tree image was generated with Tree Viewer. The HYR1 protein secondary structure was predicted using the PSIPRED protein structure prediction server. The structural image of the HYR1 protein was created using the PyMOL molecular viewer. All student t-tests were performed using JMP8 (SAS Institute Inc. 2007. *<Title>*. Cary, NC: SAS Institute Inc.).

### Confocal microscopy

Confocal images were taken with Zeiss LSM510 or Zeiss LSM5 DUO using a C-Apochromat 40X (NA = 1.2) water immersion objective lens. H_2_DCFDA ester was excited at 488 nm and fluorescence was detected using a 505–550 nm band pass filter. Calcofluor white was excited at 405 nm and detected using 420–470 nm band pass filter. Cerulean was excited at 458 nm and detected using a 475 long pass filter. We also used transmitted light and reflected light for some confocal experiments.

## Supporting Information

Figure S1 Successful deletion of the *HYR1* via homologous recombination of a single insert. (A) Diagram of strategy used for homologous recombination of *HYR1*. The arrow depicts directionality of gene MMG_07460.6, and FS stands for flanking sequence. HygR is the hygromycin phosphotransferase gene (HPH) that confers resistance to organisms that express it. Physical positions of the gene and flanking regions (from supercontig 20) are shown above the diagram. Bottom diagram shows the gene deletion construct that was PCR-ed and linked via adapters. Purple arrows indicate primer sites for determining insertion site (result shown in C). The bottom-most line indicates *HindIII* cut sites for the Southern blot, and positioning of the HPH probe. (B) External flanking region PCR indicates the insert is located in the correct position in the genome (lane loading from left to right: *Δhyr1* B25, *Δhyr1* B33, *Δhyr1* B54, ectopic B40). The size product is the expected ∼1.5kb, as based upon the primer positions in A. Gene specific primers indicate that the knockout mutant does not have *HYR1* gene. HPH specific primers indicate the HPH inserted in the genome. (C) Southern blot indicates a single insertion of the construct in the *Δhyr1* mutants. (D) Diagram of the construct used to complement the *Δhyr1* mutant; the cerulean fluorescent protein (CFP) is driven by the native *MoHYR1* promoter and linked the N-terminus of the *MoHYR1* gene. (E) Southern blot on the complemented mutant line *hyr1* -C probed with the *MoHYR1* gene, which revealed four insertions.
(1.25 MB TIF)

Figure S2 *Δhyr1* cannot grow at increased levels of hydrogen peroxide. (A) *Δhyr1* (B25, B33) growth was inhibited at increased levels of hydrogen peroxide (top  =  0mM; middle  =  5mM; bottom  =  10mM) compared to the complemented strain (hyr1- C), wild type (70-15) and Ectopic (B40, B60). (B) Quantification (dry weight) of samples grown in hydrogen peroxide. This experiment was repeated in triplicate with similar results. Different letters over the bars indicate a significant difference as determined by a student's t-test and a p-value of < 0.05.
(1.90 MB TIF)

Figure S3 *Δhyr1* accumulated similar levels of ROS to wild type *in vitro*. Hyphae of wild type and *Δhyr1* were grown on complete media plates and stained with nitroblue tetrazolium (NBT) and exhibited similar staining. A, B, C, and D are microscope images of panels E and F. A, C, and E represent *Δhyr1* (B25) and B, D, F represent wild type (70-15). Scale bars  =  100 μm.
(3.72 MB TIF)

Figure S4 *nox1* and *nox2* mutants have same ROS production with wild type on plant 24hpi. A loss of NADPH oxidases in *M. oryzae* does not appear to have a significant effect on ROS haloes. (A-F) Confocal images of the *nox1, nox2* and wild type parent lines stained with Calcofluor White (CW) for cell wall visualization and the ROS detector H_2_DCFDA. The left-most panels show multiple spores and appressoria, while the right-hand panels focus on a representative appressorium (bottom-left: H_2_DCFDA, bottom-right: CW, top: merge). (G) Graphical representation of the data collected in A showing no significant difference between ROS haloes amongst the strains. Experiments were repeated three times with similar results. Different letters over the bars indicate a significant difference as determined by a student's t-test and a p-value of < 0.05. Scale bar =  10μm.
(1.08 MB TIF)

Figure S5 *Δhyr1* displays similar levels of ROS to wild type immediately after inoculation. (A) ROS signals are detected in barley leaves 1 hpi with either the *Δhyr1* mutants or the wild type strain. *Δhyr1* mutants did not show a defect compared to wild type. Leaves treated with pathogens are significantly brighter than untreated leaves. (B) Quantification of ROS signal intensity reveals a significant difference between inoculated and untreated barley leaves. This experiment was repeated in triplicate with similar results. Different letters over the bars indicate a significant difference as determined by a student's t-test, and a p-value of < 0.05. Images are taken with confocal microscope. Scale bar  =  20 μm.
(0.77 MB TIF)

Table S1 *HYR1* amino acid sequence of *M. oryzae* is most closely related to *N. crassa*. Percent identities and similarities were determined using BlastP for ten filamentous fungi, one yeast and one mammal.
(0.01 MB XLSX)

Table S2 Primers pairs used to generate the HYR1 deletion construct and to test the targeted deletions.
(0.01 MB XLSX)

Table S3 Primers pairs used in real-time qRT-PCR experiments.
(0.01 MB XLSX)
