# Supplementary figures and images for: HYR1-Mediated Detoxification of Reactive Oxygen Species Is Required for Full Virulence in the Rice Blast Fungus
Source: PLoS Pathog. 2011 Apr 14;7(4):e1001335. doi: 10.1371/journal.ppat.1001335 (PMC3077360; doi:10.1371/journal.ppat.1001335)

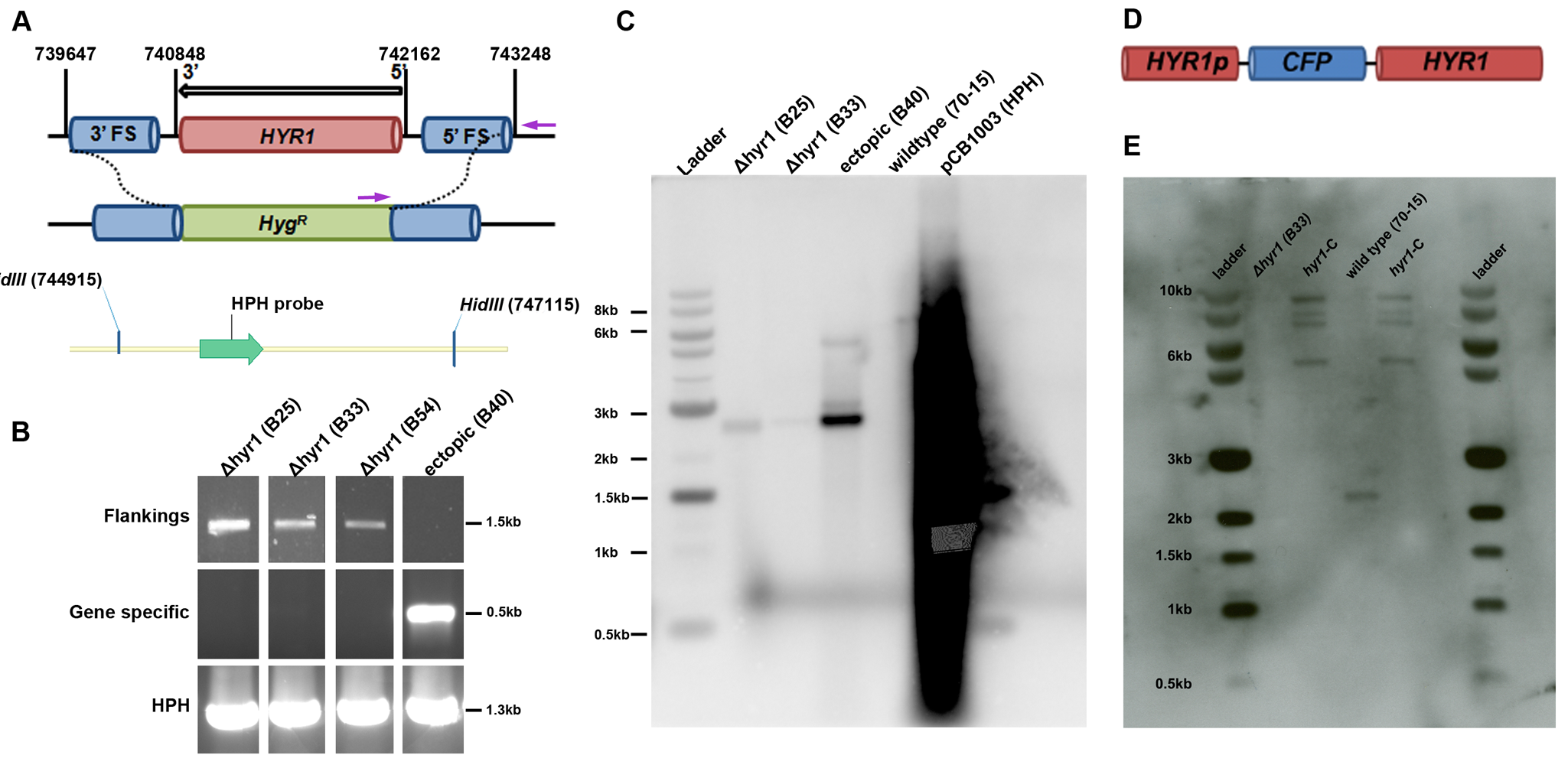

Supplement: Figure S1 — Successful deletion of the HYR1 via homologous recombination of a single insert. (A) Diagram of strategy used for homologous recombination of HYR1. The arrow depicts directionality of gene MMG_07460.6, and FS stands for flanking sequence. HygR is the hygromycin phosphotransferase gene (HPH) that confers resistance to organisms that express it. Physical positions of the gene and flanking regions (from supercontig 20) are shown above the diagram. Bottom diagram shows the gene deletion construct that was PCR-ed and linked via adapters. Purple arrows indicate primer sites for determining insertion site (result shown in C). The bottom-most line indicates HindIII cut sites for the Southern blot, and positioning of the HPH probe. (B) External flanking region PCR indicates the insert is located in the correct position in the genome (lane loading from left to right: Δhyr1 B25, Δhyr1 B33, Δhyr1 B54, ectopic B40). The size product is the expected ∼1.5kb, as based upon the primer positions in A. Gene specific primers indicate that the knockout mutant does not have HYR1 gene. HPH specific primers indicate the HPH inserted in the genome. (C) Southern blot indicates a single insertion of the construct in the Δhyr1 mutants. (D) Diagram of the construct used to complement the Δhyr1 mutant; the cerulean fluorescent protein (CFP) is driven by the native MoHYR1 promoter and linked the N-terminus of the MoHYR1 gene. (E) Southern blot on the complemented mutant line hyr1 -C probed with the MoHYR1 gene, which revealed four insertions. (1.25 MB TIF) [file ppat.1001335.s001.tif]

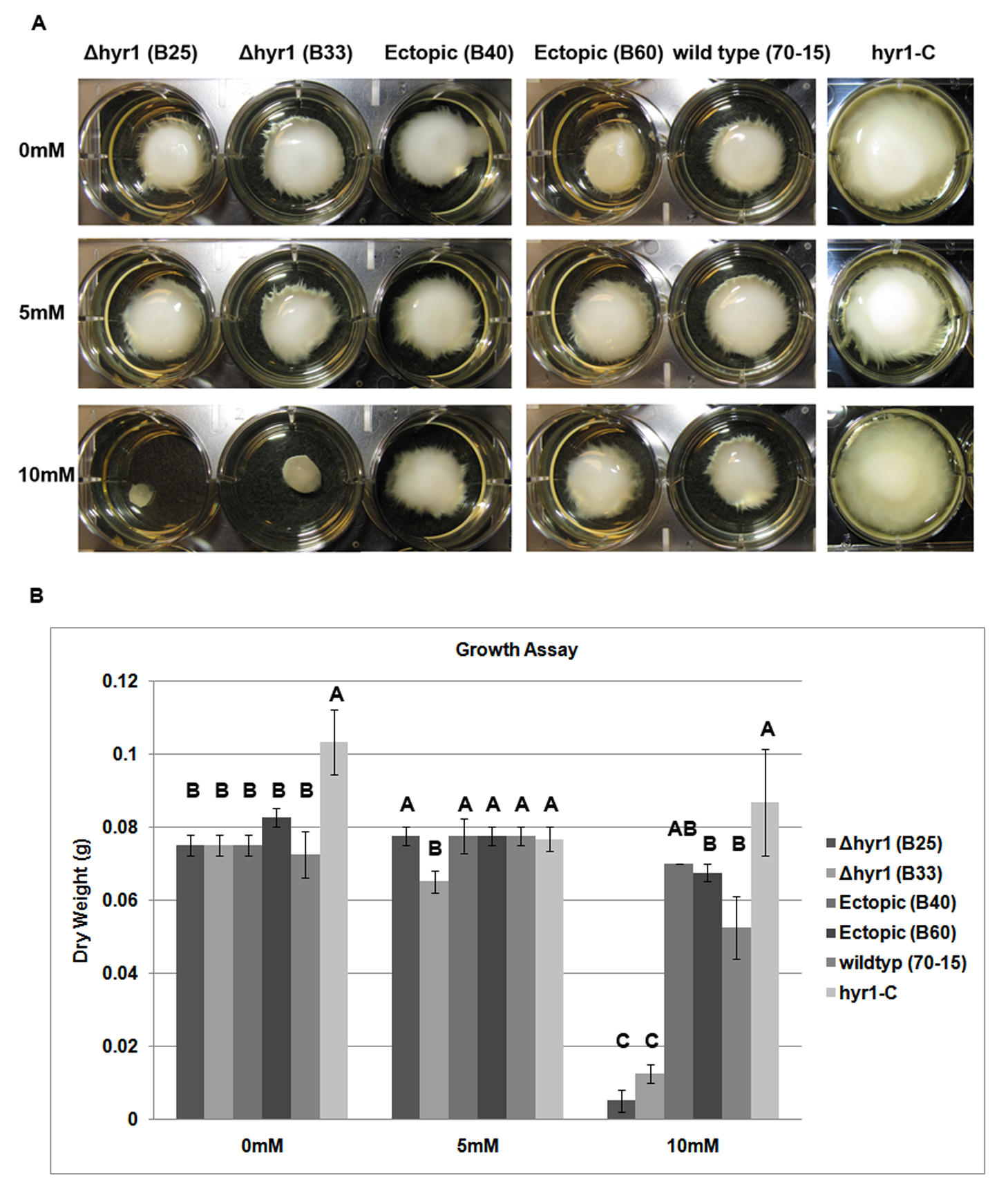

Supplement: Figure S2 — Δhyr1 cannot grow at increased levels of hydrogen peroxide. (A) Δhyr1 (B25, B33) growth was inhibited at increased levels of hydrogen peroxide (top = 0mM; middle = 5mM; bottom = 10mM) compared to the complemented strain (hyr1- C), wild type (70-15) and Ectopic (B40, B60). (B) Quantification (dry weight) of samples grown in hydrogen peroxide. This experiment was repeated in triplicate with similar results. Different letters over the bars indicate a significant difference as determined by a student's t-test and a p-value of < 0.05. (1.90 MB TIF) [file ppat.1001335.s002.tif]

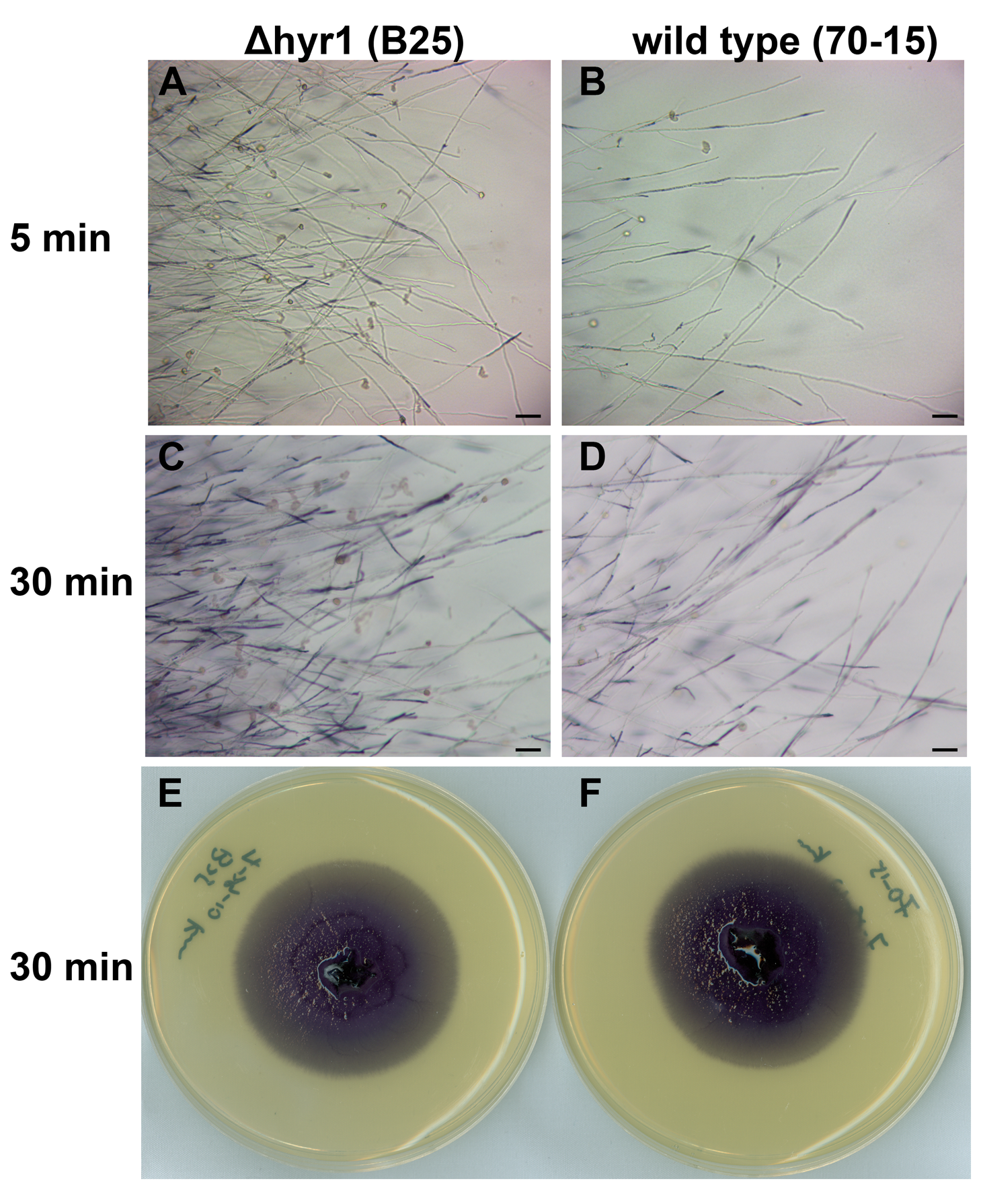

Supplement: Figure S3 — Δhyr1 accumulated similar levels of ROS to wild type in vitro. Hyphae of wild type and Δhyr1 were grown on complete media plates and stained with nitroblue tetrazolium (NBT) and exhibited similar staining. A, B, C, and D are microscope images of panels E and F. A, C, and E represent Δhyr1 (B25) and B, D, F represent wild type (70-15). Scale bars = 100 μm. (3.72 MB TIF) [file ppat.1001335.s003.tif]

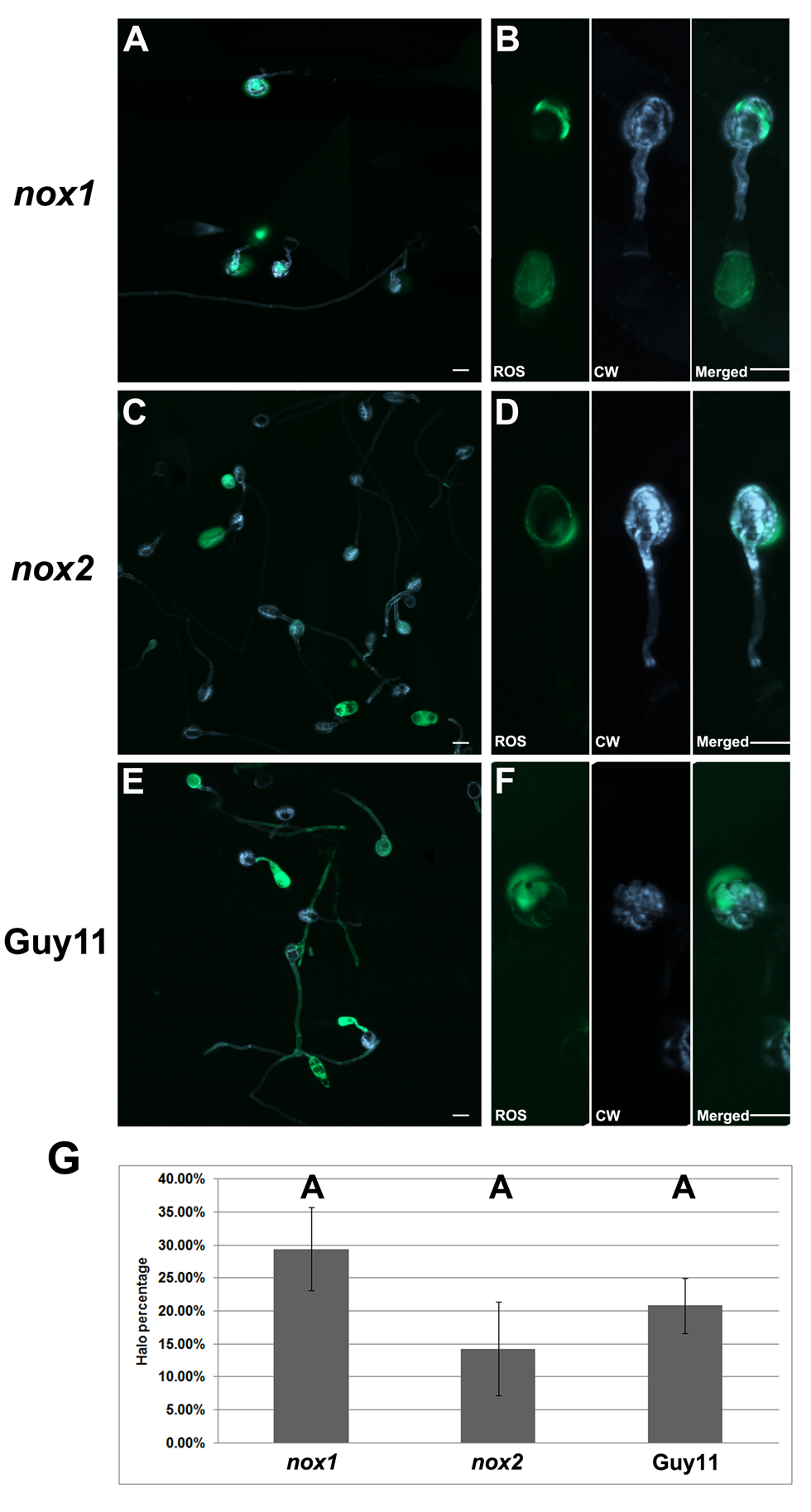

Supplement: Figure S4 — nox1 and nox2 mutants have same ROS production with wild type on plant 24hpi. A loss of NADPH oxidases in M. oryzae does not appear to have a significant effect on ROS haloes. (A-F) Confocal images of the nox1, nox2 and wild type parent lines stained with Calcofluor White (CW) for cell wall visualization and the ROS detector H2DCFDA. The left-most panels show multiple spores and appressoria, while the right-hand panels focus on a representative appressorium (bottom-left: H2DCFDA, bottom-right: CW, top: merge). (G) Graphical representation of the data collected in A showing no significant difference between ROS haloes amongst the strains. Experiments were repeated three times with similar results. Different letters over the bars indicate a significant difference as determined by a student's t-test and a p-value of < 0.05. Scale bar = 10μm. (1.08 MB TIF) [file ppat.1001335.s004.tif]

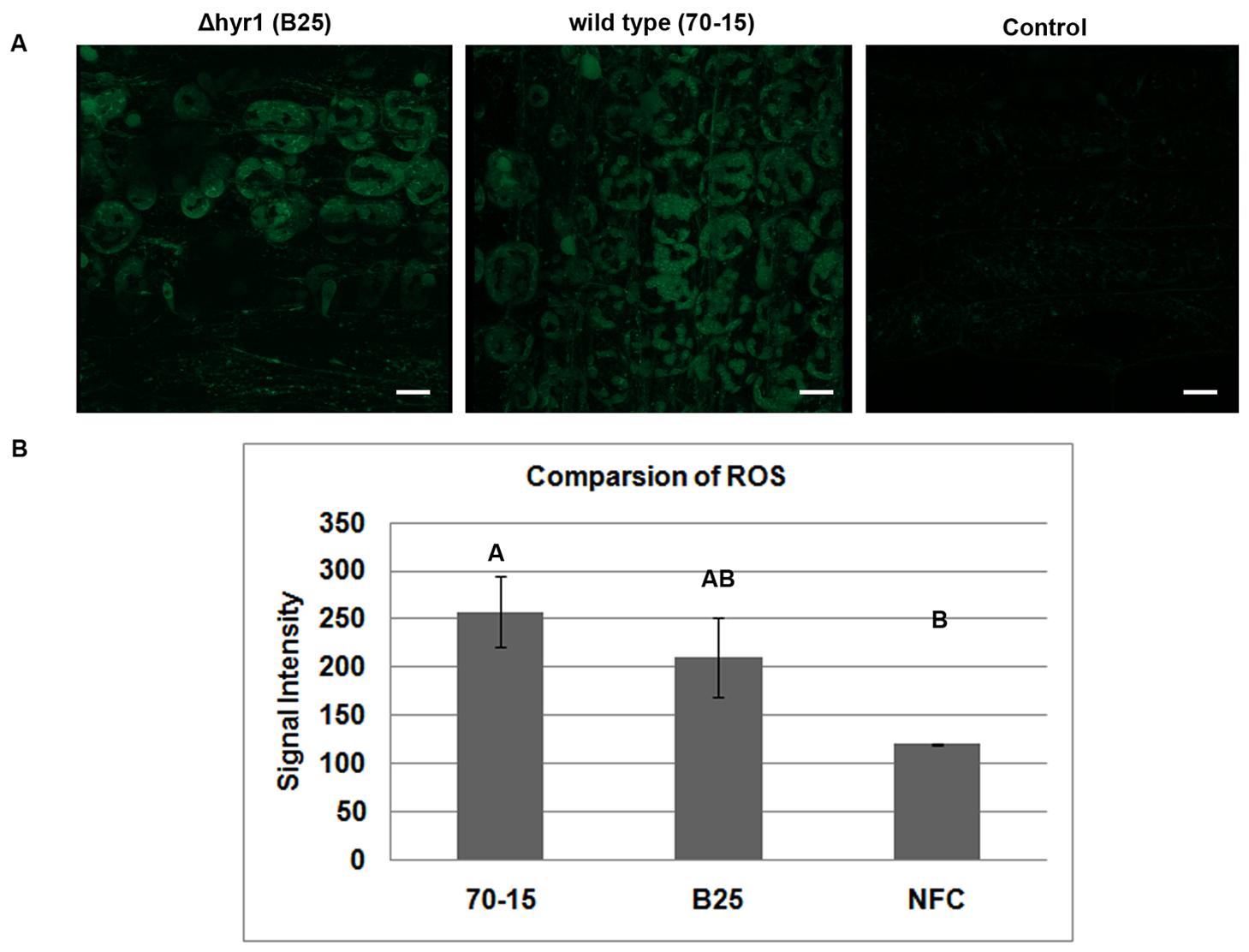

Supplement: Figure S5 — Δhyr1 displays similar levels of ROS to wild type immediately after inoculation. (A) ROS signals are detected in barley leaves 1 hpi with either the Δhyr1 mutants or the wild type strain. Δhyr1 mutants did not show a defect compared to wild type. Leaves treated with pathogens are significantly brighter than untreated leaves. (B) Quantification of ROS signal intensity reveals a significant difference between inoculated and untreated barley leaves. This experiment was repeated in triplicate with similar results. Different letters over the bars indicate a significant difference as determined by a student's t-test, and a p-value of < 0.05. Images are taken with confocal microscope. Scale bar = 20 μm. (0.77 MB TIF) [file ppat.1001335.s005.tif]
